# Understanding barriers to fruit and vegetable intake in the Australian Longitudinal Study of Indigenous Children: a mixed-methods approach

**DOI:** 10.1017/S1368980016003013

**Published:** 2016-11-29

**Authors:** Katherine Ann Thurber, Cathy Banwell, Teresa Neeman, Timothy Dobbins, Melanie Pescud, Raymond Lovett, Emily Banks

**Affiliations:** 1 National Centre for Epidemiology and Population Health, Research School of Population Health, The Australian National University, 62 Mills Road, Acton, ACT 2601, Australia; 2 Statistical Consulting Unit, The Australian National University, Acton, ACT, Australia; 3 National Drug & Alcohol Research Centre, University of New South Wales, Randwick, NSW, Australia; 4 RegNet School of Regulation and Global Governance, The Australian National University, Acton, ACT, Australia; 5 The Australian Prevention Partnership Centre, Sax Institute, Ultimo, NSW, Australia

**Keywords:** Diet, food and nutrition, Child health, Holistic health, Health behaviour

## Abstract

**Objective:**

To identify barriers to fruit and vegetable intake for Indigenous Australian children and quantify factors related to these barriers, to help understand why children do not meet recommendations for fruit and vegetable intake.

**Design:**

We examined factors related to carer-reported barriers using multilevel Poisson models (robust variance); a key informant focus group guided our interpretation of findings.

**Setting:**

Eleven diverse sites across Australia.

**Subjects:**

Australian Indigenous children and their carers (*N* 1230) participating in the Longitudinal Study of Indigenous Children.

**Results:**

Almost half (45 %; *n* 555/1230) of carers reported barriers to their children’s fruit and vegetable intake. Dislike of fruit and vegetables was the most common barrier, reported by 32·9 % of carers; however, we identified few factors associated with dislike. Carers were more than ten times less likely to report barriers to accessing fruit and vegetables if they lived large cities *v*. very remote areas. Within urban and inner regional areas, child and carer well-being, financial security, suitable housing and community cohesion promoted access to fruit and vegetables.

**Conclusions:**

In this national Indigenous Australian sample, almost half of carers faced barriers to providing their children with a healthy diet. Both remote/outer regional carers and disadvantaged urban/inner regional carers faced problems accessing fruit and vegetables for their children. Where vegetables were accessible, children’s dislike was a substantial barrier. Nutrition promotion must address the broader family, community, environmental and cultural contexts that impact nutrition, and should draw on the strengths of Indigenous families and communities.

Poor diet is the leading preventable risk factor for poor health in Australia, estimated to account for more than 10 % of the total burden of disease^(^ ^1^ ^)^, including through its association with conditions including cardiovascular and circulatory diseases, cancer and diabetes. The disease burden attributable to poor diet is estimated to be nearly double (19 %) in the Indigenous population; four of the seven leading risk factors contributing to the gap in health between the Indigenous and non-Indigenous populations relate to diet (obesity, high blood cholesterol, high blood pressure, low fruit and vegetable intake)^(^ ^2^ ^)^.

Australian guidelines recommend that children aged 4–8 years consume 1½ servings of fruit and 4½ servings of vegetables daily, increasing to 2 and 5 servings daily, respectively, for children aged 9–11 years^(^ ^3^ ^)^. Data from a 2012–2013 national survey indicated that while 78 % of Indigenous children met recommendations for fruit intake, only 16 % met recommendations for vegetable intake^(^ ^4^ ^)^, consistent with statistics for non-Indigenous Australian children^(^ ^5^ ^)^. Increasing fruit and particularly vegetable intake by Indigenous children could decrease the burden of disease. However, there is limited evidence on the effectiveness of existing programmes and policy to improve Indigenous nutrition^(^ ^6^ ^)^.

Indigenous Australians have a diversity of cultures and live in varied environments^(^ ^7^ ^)^. About 57 % of the Indigenous population lives in major cities or inner regional areas (approximately 380 800 people) and 21 % in remote or very remote settings (approximately 142 900 people), compared with 90 % and 2 % of the non-Indigenous population, respectively^(^ ^8^ ^)^. On average, Indigenous Australians have lower socio-economic status than non-Indigenous Australians, and are more likely to live in homes that are overcrowded or have infrastructure problems^(^ ^9^ ^,^ ^10^ ^)^ and to live in areas of socio-economic deprivation^(^ ^11^ ^)^.

Food choice is complex and influenced by a broad range of factors^(^ ^12^ ^–^ ^15^ ^)^. The ability to purchase fruit and vegetables is influenced by their accessibility, affordability and availability; in Australia, basic healthy food items are less likely to be available and more likely to be more expensive and of lower quality in remote areas compared with urban centres^(^ ^16^ ^–^ ^18^ ^)^. Food purchasing behaviour is strongly associated with socio-economic status and is also shaped by individual preferences and cultural factors^(^ ^12^ ^,^ ^13^ ^,^ ^17^ ^,^ ^19^ ^–^ ^22^ ^)^. Connection and commitment to extended family and community members, and community organisations and events have been identified as factors supporting Indigenous well-being^(^ ^23^ ^,^ ^24^ ^)^. For example, cultural values and norms about reciprocity and generosity encourage Indigenous families to share food and resources with others^(^ ^25^ ^)^; many households often feed extra people^(^ ^12^ ^,^ ^26^ ^,^ ^27^ ^)^ and ‘humbugging’, wherein relatives or friends request money or resources, is a common practice in some communities^(^ ^25^ ^,^ ^28^ ^)^. Traditional knowledge and food systems are also understood to promote Indigenous nutrition^(^ ^29^ ^)^; however, these were disrupted through colonisation and its lasting impacts on the Indigenous population, resulting in profound changes to Indigenous food practices^(^ ^13^ ^,^ ^26^ ^,^ ^30^ ^,^ ^31^ ^)^. Contemporarily, market foods are estimated to generally constitute the majority of food intake by Indigenous Australians, with ‘bush tucker’ (hunted and cultivated traditional foods) a minor contributor to intake^(^ ^29^ ^,^ ^32^ ^–^ ^34^ ^)^.

Overall, Indigenous children disproportionately face barriers to fruit and vegetable intake compared with non-Indigenous children, given the population distribution, lower socio-economic status, and unique historical, political and cultural context. Qualitative research in both remote and urban settings has depicted how broader social, cultural and environmental factors influence Indigenous Australians’ food choice^(^ ^12^ ^,^ ^13^ ^,^ ^17^ ^,^ ^19^ ^,^ ^20^ ^)^. The purpose of the current work was to quantify factors related to perceived barriers to fruit and vegetable intake for Indigenous Australian children, in order to understand why children do not meet recommendations for intake. We have focused on identifying protective (rather than risk) factors that facilitate children’s consumption of fruit and vegetables. Within this, we explore variation between urban, regional and remote settings, given the vast differences in food environments, population characteristics and cultural contexts.

## Methods

### Mixed methods

The current study analyses quantitative data from the Longitudinal Study of Indigenous Children (LSIC) and qualitative data from a key informant focus group. Rather than relying on quantitative epidemiological analyses on their own, we conducted a focus group as part of our research protocol to guide data interpretation^(^ ^35^ ^)^. This mixed-methods approach^(^ ^36^ ^)^ facilitates a more holistic analysis, considering multiple perspectives and drawing on multiple ways of sharing knowledge^(^ ^37^ ^,^ ^38^ ^)^, and enhances the interpretation and contextualisation of findings^(^ ^35^ ^)^. Our approach was guided by Bronfenbrenner and Morris’ social ecological model^(^ ^39^ ^)^ and a conceptual framework applied within the Pro Children project^(^ ^40^ ^,^ ^41^ ^)^.

### Study population

The LSIC is a national study managed by the Australian Government’s Department of Social Services^(^ ^42^ ^)^. Purposive sampling was used to recruit Aboriginal and Torres Strait Islander children from eleven diverse sites across Australia. The survey design and the implications for analysis have been described elsewhere^(^ ^42^ ^,^ ^43^ ^)^. Indigenous Research Administration Officers (RAOs) conduct annual face-to-face interviews with children and their primary carer (the child’s mother in the majority of cases, or sometimes the father, a relative or other guardian). The present analysis is based on data collected in 2013, in Wave 6 of the LSIC. The primary carer reported all data included in the analysis, with the exception of children’s BMI, which was calculated based on height and weight measurements taken by RAOs, and remoteness and area-level disadvantage, which were derived from participants’ addresses.

### Focus group methods

In February 2015, the lead author (K.A.T.), a non-Indigenous woman, conducted a focus group with the RAOs currently conducting LSIC interviews (*n* 12/12). As all RAOs are Indigenous and most live in the sites in which they conduct interviews, we considered them key informants in these communities, holding pertinent contextual knowledge^(^ ^35^ ^)^.

The focus group was semi-structured to facilitate the sharing of stories and to allow new issues to emerge throughout the discussion^(^ ^44^ ^)^; the participants guided the depth and focus of discussion on each topic. During the focus group, K.A.T. presented preliminary descriptive analysis of the relevant LSIC data, including: the distribution of carer-reported fruit and vegetable intake by children, overall and by remoteness; carer-reported barriers to children’s fruit and vegetable intake, overall and by remoteness; examples of carers’ free-text responses about why their children did not eat more fruit and vegetables; and examples of carers’ free-text responses about how they encouraged their children to eat more fruit and vegetables. For each item, K.A.T. asked RAOs to comment if this was consistent with the experience they had in the site in which they worked, if they thought the findings were important and what they thought the findings meant. RAOs were encouraged to share stories and experiences from the field.

K.A.T. conducted, transcribed and analysed the focus group interview, which was audio-recorded with participants’ content. The LSIC team reviewed and approved the transcript; a follow-up discussion was held with the RAOs in 2016 to discuss the interpretation of final results and their implications.

### Variables in the quantitative analysis

#### Exposures

##### Child factors

We examined children’s age at the time of survey, sex, and identification as Aboriginal, Torres Strait Islander or both. We also examined indicators of the child’s well-being: general health, social and emotional well-being (low/moderate *v*. high risk of social and emotional behavioural difficulties according to the Strengths and Difficulties Questionnaire^(^ ^45^ ^,^ ^46^ ^)^), and BMI category^(^ ^47^ ^,^ ^48^ ^)^.

##### Family factors

We examined objective and subjective measures of families’ financial situations: carer’s relationship status, weekly household income, financial strain (run out of money before payday/spending more than earning; enough money to get through to the next payday; can save a bit/a lot), serious worries about money in past year, food insecurity (going without meals because of a lack of money) in the past year, and carer’s highest qualification and employment status. We examined indicators of resource sharing: being humbugged in the past year, frequency of feeding others who don’t live at home and pressures to support others in the community.

We also examined carers’ general health and social and emotional well-being (low or high distress, based on a social and emotional well-being index^(^ ^49^ ^)^) and the number of negative major life events experienced by the family in the past year.

We examined whether the family had an evening meal together in the past week and carers’ perceptions of the importance of passing on cultural knowledge to their children about bush tucker, hunting and fishing. We also examined household size and housing problems (home has felt too crowded in past year; moved house in past year; problems with fridge and/or cooking facilities; major electrical problems at home; security problems at home – major problems with locks, windows, doors or screens).

##### Area-level factors

Geographical remoteness in the LSIC is measured using the Level of Relative Isolation scale^(^ ^50^ ^)^. Areas are categorised as having no, low, moderate, high or extreme isolation; these categories correspond to major cities, larger regional centres, smaller regional centres far from large cities and communities/settlements generally with a predominantly Indigenous population, respectively. For stratified analyses, we classified areas with no or low isolation as urban/inner regional (urban/IR) and areas with moderate or high/extreme isolation as remote/outer regional (remote/OR).

Area-level disadvantage was measured using the Index of Relative Indigenous Socioeconomic Outcomes (IRISEO)^(^ ^51^ ^)^, an index calculated specifically for Indigenous Australians based on nine measures of socio-economic status. We categorised areas as having the highest level of advantage (IRISEO 8–10), mid-level advantage (IRISEO 4–7) and the lowest level of advantage (IRISEO 1–3).

Carers also reported if they experienced a problem with racially motivated violence, alcohol misuse, and break-ins or theft in their community.

See the online supplementary material, Supplemental File 1, for more details on exposure variables.

#### Outcome

All carers were asked if they would like their children to eat more fruit and/or vegetables. Carers responding that they wanted their children to eat more were asked to select up to two barriers to their child’s fruit and/or vegetables intake: ‘child doesn’t like them or refuses to eat them’, ‘too expensive’, ‘not readily available (e.g. shop doesn’t have enough)’, ‘poor quality of fresh produce’, ‘issue with transport (e.g. shop too far away or no car)’, ‘no food preparation area or storage’, ‘don’t know’ or ‘other’. Carers reporting barriers related to accessibility, affordability and availability (‘too expensive’, ‘not readily available’, ‘poor quality’, ‘issue with transport’, ‘no food preparation area or storage’) were categorised as perceiving accessibility-related barriers. Carers responding that their children ‘had enough’ fruit and vegetables were considered to perceive no barriers to intake. Carers who responded ‘don’t know’ (0·4 %, *n* 5/1239) or who specified a different answer (0·3 %, *n* 4/1239) were excluded.

To ensure that our outcome variable was meaningful, we validated that carers’ perceptions of barriers reflected low fruit and vegetable intake by their children, and that the relationships of exposures to carers’ perception of barriers were consistent with the relationships of these exposures to children’s low vegetable intake. This validation was conducted within the sample of children (*n* 502/1230) who had data on dietary intake (see online supplementary material, Supplemental File 2).

### Analytical methods

Rather than looking at factors relating to carers’ perception of any barriers, we separately examined factors related to accessibility barriers and factors related to children’s dislike of fruit and vegetables, as the factors related to these two barrier types might vary. It is difficult to interpret dislike if children have limited access to fruit and vegetables, or if they only have access to fruit and vegetables that are of poor quality, so we examined factors related to children’s dislike in the sample restricted to those without any accessibility barriers.

We calculated prevalence ratios (PR) using multilevel Poisson models with robust variance. Children’s geographic cluster was included as a level variable to account for the within-cluster correlation resulting from the LSIC’s survey design. We first adjusted these models for age group and sex only. We expected that remoteness might confound these relationships, given known variation in food availability and sociocultural context across levels of remoteness, so we repeated the models with additional adjustment for remoteness and examined how this altered relationships. If findings indicated potential residual confounding by remoteness, we repeated analyses separately in the urban/IR and remote/OR groups. We tested if the exposure–outcome relationships varied between urban/IR and remote/OR environments by repeating the models in the whole sample including an interaction term between the dichotomous remoteness variable and the exposure, with *P*_interaction_<0·05 indicating a significant difference.

Analysis of the qualitative data from the focus group guided our interpretation of quantitative findings^(^ ^35^ ^)^. The qualitative data were analysed thematically^(^ ^52^ ^)^. Inductive and deductive codes were developed based on our open-ended questions and refined as new information was found. Coded textual segments were grouped into categories and sub-categories which were then developed into themes^(^ ^53^ ^)^ and further refined and cross-checked through discussions within the team. The qualitative findings reported in the present manuscript reflect the outcomes of the group discussion and the unique viewpoints of RAOs working in different settings.

### Ethics

The LSIC survey was conducted with ethical approval from the Departmental Ethics Committee of the Australian Commonwealth Department of Health and from relevant Ethics Committees in each state and territory, including relevant Aboriginal and Torres Strait Islander organisations. The Australian National University’s Human Research Ethics Committee granted ethics approval for the quantitative analysis of LSIC data in October 2011 (protocol number 2011/510); approval to conduct the focus group and for subsequent engagement with key informants was granted in 2015 and 2016, respectively.

## Results

### Profile of the sample

Interviews were conducted with 1239 carers in LSIC Wave 6, of whom 1230 provided data on barriers to their children’s fruit and vegetable intake. Children in the younger cohort were aged 4–6 years and children in the older cohort were aged 7–10 years; 614 female and 616 male children were included in the sample. The majority of families in the sample were living in urban/IR areas, 78·7 % (*n* 968/1230), with 21·3 % (*n* 262/1230) living in remote/OR areas. Characteristics of the participating families are presented in Table 1.

**Table 1:** Profile of families participating in the Australian Longitudinal Study of Indigenous Children (LSIC), Wave 6, 2013

	Remote/OR (*N* 262)		Urban/IR (*N* 968)		Total (*N* 1230)	
	%	*n*/*N*	%	*n*/*N*	%	*n*/*N*
Carer is partnered	58·0	152/262	54·7	529/968	55·4	681/1230
Carer has education Year 12 or further	39·5	92/233	44·4	399/899	43·4	491/1132
Household income ≥$AU 600/week	47·3	104/220	63·7	581/912	60·5	685/1132
Family has not been humbugged in past year	60·2	157/261	75·8	733/967	72·5	890/1228
No problem with pressure to support others in the community	51·0	126/247	79·8	700/877	73·5	826/1124
Household size ≥6 members	52·3	137/262	35·4	343/968	39·0	480/1230
Households with working fridge and cooking facilities	82·7	143/173	95·0	667/702	92·6	810/875
Living in areas in highest tertile of advantage	3·8	10/262	25·9	251/968	21·2	261/1230
Older cohort only (*N* 495)	Mean	95 % CI	Mean	95 % CI	Mean	95 % CI
Number of usual daily servings of fruit	2·1	1·9, 2·3	2·1	2·0, 2·2	2·1	2·0, 2·2
*n*		99		394		493
Number of usual daily servings of vegetables	1·8	1·6, 2·1	1·9	1·7, 2·0	1·9	1·8, 2·0
*n*		99		396		495

The sample includes those with data on barriers. Data on intake of fruit and vegetables were recorded for the older cohort (children aged 7–10 years) only. Sample size varies due to missing data on the exposures of interest. Urban/inner regional (urban/IR) areas were defined as those with no or low isolation (major cities and larger regional centres) and remote/outer regional (remote/OR) areas as those with moderate or high/extreme isolation (smaller regional centres far from large cities and communities/settlements generally with a predominantly Indigenous population).

On average, carers reported that children in the older cohort consumed 2·1 servings of fruit daily, with about 80 % meeting the recommended daily intake for their age group. Vegetable intake was much lower in the sample, with less than 5 % of children meeting their recommended daily intake; on average, carers reported that children consumed 1·9 servings of vegetables daily. We did not observe significant variation in intake between remote/OR and urban/IR settings.

### Reported barriers

Almost half of carers (45·1 %; *n* 555/1230) reported a barrier to their child’s fruit and vegetable intake (Table 2); 17·5 % (*n* 215) reported barriers to vegetable intake only, 4·1 % (*n* 51) reported barriers to fruit intake only, and 23·5 % (*n* 289) reported barriers to both fruit and vegetable intake. In the following analyses, we have examined barriers to fruit and vegetable intake combined.

**Table 2:** Barriers to children’s fruit and vegetable consumption reported by carers in the Australian Longitudinal Study of Indigenous Children (LSIC), Wave 6, 2013

	Carer-reported barriers to children’s fruit and vegetable intake					
	Remote/OR		Urban/IR		Total	
	%	*n*/*N*	%	*n*/*N*	%	*n*/*N*
No barriers to intake	51·9	136/262	55·7	539/968	54·9	675/1230
Children’s dislike	24·8	65/262	35·1	340/968	32·9	405/1230
Any accessibility barriers	24·0	63/262	2·9	28/968	7·4	91/1230
Too expensive	11·5	30/262	2·2	21/968	4·1	51/1230
Not available	13·7	36/262	≤0·3	≤3/968	≤3·2	≤39/1230
Poor quality	9·5	25/262	≤0·3	≤3/968	≤2·3	≤28/1230
Transport issues	1·5	4/262	0·5	5/968	0·7	9/1230
No preparation or storage area	≤1·1	≤3/262	≤0·3	≤3/968	≤0·5	≤6/1230
Other	2·7	7/262	7·7	75/968	6·7	82/1230
Don’t know or refused	≤1·1	≤3/262	2·7	26/968	≤2·4	≤29/1230

Remote/OR, remote/outer regional; urban/IR, urban/inner regional. Carers who reported any barriers to their children’s fruit and vegetable intake were allowed to select up to two specific barriers from the provided response options. The table includes data on both of the carers’ responses if they reported two different barriers; thus, the sum of all responses sums to more than the total percentage reporting any barrier.

Children’s dislike of fruit and/or vegetables was the most common barrier, reported by 32·9 % (*n* 405/1230) of carers. An additional 7·4 % of carers reported barriers related to accessibility: 4·1 % (*n* 51/1230) said fruit and vegetables were too expensive, ≤3·2 % (*n* ≤ 39/1230) said they were not available, ≤2·3 % (*n*≤ 28/1230) said they were of poor quality, 0·7 % (*n* 9/1230) reported issues with transport and ≤0·5 % (*n*≤6/1230) said that food preparation or storage areas were not available. Few carers (1·2 %; *n* 15/1230) reported barriers related to both dislike and accessibility.

An additional 6·7 % of carers specified other reasons (*n* 82/1230 carers providing ninety-four total responses), with the main themes of: fussy eating and children making their own choices (*n* 20); carers’ cooking and eating habits, including time constraints (*n* 14); the preparation or appearance of fruit and vegetables (*n* 9); dietary restrictions due to disability or health conditions (*n* 9); the type or variety of fruit and vegetables available (*n* 8); ‘running out’ of fruit and vegetables at home quickly, or not purchasing enough (*n* 7); and competition from alternatives such as junk food (*n* 6).

**Fig. 1:**
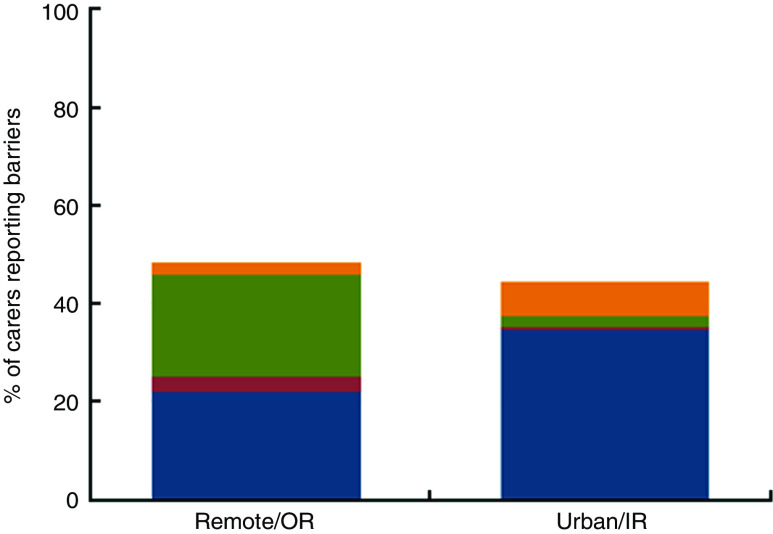
(colour online) Categories of carer-reported barriers to children’s fruit and vegetable consumption in urban/inner regional (urban/IR) and remote/outer regional (remote/OR) settings among families participating in the Australian Longitudinal Study of Indigenous Children (LSIC), Wave 6, 2013 (*N* 1230). Responses were categorised as ‘Child dislike only’ (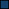) if the only barrier the carer reported was that the child did not like fruit and/or vegetables; ‘Accessibility only’ (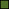) if the carer only reported barriers related to accessibility and availability (too expensive, not available, poor quality, transport issues, and no storage); ‘Accessibility + child dislike’ (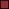) if the carer reported children’s dislike and a barrier related to accessibility or availability; or ‘Other, don’t know or refused only’ (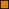)

The proportion of carers reporting any barriers to the child’s fruit and vegetable consumption was 48·1 % in remote/OR and 44·3 % in urban/IR areas. Children’s dislike of fruit and vegetables was the most common barrier in both settings, but barriers related to accessibility were more commonly reported by remote/OR carers (Table 2 and Fig. 1). In remote/OR settings, 24·8 % (*n* 65/262) of carers reported children’s dislike, 24·0 % (*n* 63/262) reported any accessibility barriers and 2·7 % (*n* 7/262) specified other reasons. In urban/IR settings, 35·1 % (*n* 340/968) of carers reported children’s dislike, 2·9 % (*n* 28/968) reported any accessibility barriers and 7·7 % (*n* 75/968) specified other reasons.

### Factors associated with accessibility barriers

Accessibility barriers were strongly associated with remoteness. The percentage of carers reporting an accessibility barrier increased from 2·0 % (*n* 7/348) in areas with the lowest level of remoteness to 33·7 % (*n* 35/104) in areas with the highest level of remoteness, corresponding to a greater than tenfold increased prevalence after adjustment for age and sex (PR=14·1, 95 % CI 4·3, 46·4). The magnitude of these differences indicated that simply adjusting for remoteness might result in residual confounding by remoteness, so we present the analyses stratified by remote/OR *v*. urban/IR status (see online supplementary material, Supplemental File 3, for unstratified results).

#### Remote/outer regional areas

Within remote/OR areas, accessibility barriers were significantly associated with indicators of family financial status, resource sharing and housing, as well as community-level factors (Table 3). Remote/OR carers were significantly less likely to report accessibility barriers if they did not experience food insecurity in the past year (20·3 *v*. 59·3 %; PR=0·6, 95 % CI 0·4, 0·9), were not humbugged in the past year (16·6 *v*. 35·6 %; PR=0·6, 95 % CI 0·4, 1·0) and did not have electrical problems at home (21·4 *v*. 64·7 %; PR=0·7, 95 % CI 0·5, 0·9). Carers who lived in communities where racially motivated violence was not a problem (23·2 *v*. 46·7 %; PR=0·8, 95 % CI 0·7, 1·0), or in areas with higher levels of advantage, were significantly less likely to report accessibility barriers. Carers who considered it important to teach their children about bush tucker were more likely to report accessibility barriers than carers who did not consider it important.

**Table 3:** Factors associated with carers perceiving accessibility-related barriers to children’s fruit and/or vegetable intake, according to remoteness, in the Australian Longitudinal Study of Indigenous Children (LSIC), Wave 6, 2013

	Remote/OR				Urban/IR			
	%	*n*/*N*	PR	95 % CI	%	*n*/*N*	PR	95 % CI
Total	24·1	63/262	–	–	2·9	28/968	–	–
Child factors								
Sex								
Male	21·6	27/125	1·00		3·7	18/491	1·00	
Female	26·3	36/137	1·03	0·78, 1·37	2·1	10/477	0·57	0·26, 1·27
Age group								
4–5 years	26·4	14/53	1·00		4·1	9/222	1·00	
6–7 years	24·1	27/112	1·10	0·74, 1·65	2·5	9/355	0·60	0·24, 1·52
8–10 years	22·7	22/97	0·85	0·57, 1·26	2·6	10/391	0·70	0·32, 1·53
Indigenous identification†,§								
Aboriginal	29·1	55/189	1·00		3·1	27/881	1·00	
Torres Strait Islander	≤6·1	≤3/49	0·45	0·08, 2·49	≤7·9	≤3/38	0·00	0·00, 0·00
Both	20·8	5/24	0·91	0·38, 2·19	≤6·1	≤3/49	0·52	0·05, 5·21
General physical health								
Poor, fair, or good	36·5	42/115	1·00		4·8	9/189	1·00	
Very good or excellent	14·3	21/147	0·62	0·38, 1·01	2·4	19/779	0·74	0·37, 1·49
Social and emotional well-being†,‡								
High risk of difficulties	31·3	20/64	1·00		7·4	16/217	1·00	
Low risk of difficulties	21·7	43/198	0·94	0·64, 1·37	1·6	12/748	0·23	0·12, 0·45
BMI category								
Overweight or obese	23·3	14/60	1·00		2·4	8/331	1·00	
Normal weight	28·4	38/134	0·75	0·53, 1·06	3·2	14/441	1·41	0·73, 2·73
Underweight	≤25·0	≤3/12	0·54	0·16, 1·81	≤50·0	≤3/6	2·00	1·01, 3·99
Family factors								
Carer’s general physical health								
Poor, fair, or good	27·8	49/176	1·00		3·6	19/529	1·00	
Very good or excellent	15·3	13/85	0·66	0·34, 1·30	2·1	9/439	0·74	0·34, 1·60
Carer’s social and emotional well-being†,‡								
High distress	15·7	8/51	1·00		7·3	14/191	1·00	
Low distress	26·3	55/209	1·55	0·69, 3·48	1·8	14/769	0·27	0·15, 0·46
Negative major life events in past year								
3–9	32·3	30/93	1·00		3·7	11/294	1·00	
<3	19·5	33/169	0·82	0·63, 1·07	2·5	17/673	0·63	0·33, 1·22
Carer is partnered†,‡								
No	20·0	22/110	1·00		4·1	18/439	1·00	
Yes	27·0	41/152	1·01	0·65, 1·58	1·9	10/529	0·41	0·22, 0·79
Weekly household income†								
<$AU 600	39·7	46/116	1·00		4·5	15/331	1·00	
≥$AU 600	9·6	10/104	0·62	0·30, 1·28	2·1	12/581	0·51	0·29, 0·91
Financial strain†,‡								
Run out of money	68·4	13/19	1·00		6·7	8/120	1·00	
Just enough money	27·5	28/102	0·82	0·48, 1·40	4·3	18/419	0·56	0·23, 1·34
Can save money	15·8	22/139	0·62	0·36, 1·07	0·5	2/425	0·07	0·02, 0·30
Worries about money in past year†,‡								
Yes	20·5	9/44	1·00		4·9	14/287	1·00	
No	25·1	54/215	1·26	0·90, 1·77	2·1	14/678	0·36	0·19, 0·68
Went without meals in past year*,†								
Yes	59·3	16/27	1·00		13·3	6/45	1·00	
No	20·3	47/231	0·56	0·36, 0·86	2·4	22/920	0·30	0·11, 0·77
Carer’s employment status								
Not employed	25·5	41/161	1·00		3·7	21/574	1·00	
Employed part-time	34·3	12/35	1·07	0·69, 1·66	2·0	4/203	0·63	0·21, 1·89
Employed full-time	14·5	9/62	0·85	0·51, 1·44	≤1·7	≤3/172	0·48	0·19, 1·17
Carer’s highest qualification								
Less than Year 12	28·4	40/141	1·00		3·6	18/500	1·00	
Year 12 and beyond	22·8	21/92	1·16	0·83, 1·61	2·0	8/399	0·64	0·28, 1·45
Humbugged in past year*,†								
Yes	35·6	37/104	1·00		6·0	14/234	1·00	
No	16·6	26/157	0·63	0·42, 0·95	1·9	14/733	0·39	0·16, 0·92
Pressured to support others in the community†								
Small or big problem	34·7	42/121	1·00		7·3	13/177	1·00	
Not a problem	14·3	18/126	0·63	0·33, 1·22	1·6	11/700	0·27	0·12, 0·62
Feed others who don’t live at home								
A few times a month or more	29·2	42/144	1·00		3·2	18/569	1·00	
Rarely or never	17·1	20/117	0·74	0·51, 1·09	2·5	10/398	0·91	0·46, 1·82
Had evening meal as a family in past week								
No	≤15·8	≤3/19	1·00		**≤7**·**1**	≤3/42	1·00	
Yes	25·0	60/240	0·74	0·34, 1·62	2·8	26/924	0·48	0·18, 1·26
Cultural knowledge about bush tucker*,†								
Not important	12·6	12/95	1·00		1·8	13/715	1·00	
Somewhat important	25·0	25/100	1·17	0·68, 2·00	4·1	7/170	1·77	0·78, 4·01
Very important	38·8	26/67	1·73	0·99, 3·01	9·8	8/82	3·79	1·69, 8·50
Total number of people in household								
2–5	22·4	28/125	1·00		1·9	12/625	1·00	
≥6	25·5	35/137	0·79	0·53, 1·16	4·7	16/343	1·80	0·71, 4·53
House felt too crowded in past year†,‡								
Yes	27·0	10/37	1·00		8·0	9/113	1·00	
No	23·3	52/223	1·09	0·51, 2·36	2·2	19/850	0·33	0·17, 0·67
Moved house in past year								
Yes	28·2	11/39	1·00		3·1	6/194	1·00	
No	23·1	51/221	0·92	0·71, 1·21	2·9	22/769	1·23	0·49, 3·05
Problem with fridge and/or cooking facilities†,‡								
Yes	30·0	9/30	1·00		14·3	5/35	1·00	
No	19·6	28/143	1·09	0·73, 1·62	2·4	16/667	0·17	0·08, 0·36
Electrical problems at home*								
Yes	64·7	11/17	1·00		7·7	4/52	1·00	
No	21·4	52/243	0·69	0·51, 0·92	2·6	24/915	0·40	0·15, 1·07
Security problems at home†								
Yes	46·7	21/45	1·00		8·9	8/90	1·00	
No	19·5	42/215	0·76	0·50, 1·14	2·3	20/877	0·38	0·16, 0·88
Area-level factors								
Racially-motivated violence*,†,‡								
Small or big problem	46·7	7/15	1·00		5·6	9/162	1·00	
Not a problem	23·2	53/228	0·83	0·69, 0·99	2·1	15/729	0·23	0·10, 0·55
Alcohol misuse†								
Small or big problem	27·5	53/193	1·00		4·9	19/386	1·00	
Not a problem	12·5	8/64	0·56	0·28, 1·13	1·6	9/552	0·40	0·17, 0·93
Break-ins or theft								
Small or big problem	27·8	35/126	1·00		3·7	17/461	1·00	
Not a problem	21·4	27/126	0·96	0·69, 1·32	2·0	9/455	0·50	0·23, 1·08
Area-level disadvantage*,†								
Least advantaged	39·3	59/150	1·00		13·8	9/65	1·00	
Mid-advantaged	≤2·9	≤3/102	0·11	0·02, 0·56	2·6	17/652	0·21	0·06, 0·76
Most advantaged	≤30·0	≤3/10	0·42	0·12, 1·45	≤1·2	≤3/251	0·06	0·01, 0·34
Remoteness								
None	–	–	–	–	2·0	7/348	1·00	
Low	–	–	–	–	3·4	21/620	1·64	0·55, 4·90
Moderate	17·7	28/158	1·00		–	–	–	–
High/extreme	33·7	35/104	2·04	0·63, 6·62	–	–	–	–

Remote/OR, remote/outer regional; urban/IR, urban/inner regional; PR, prevalence ratio. All models are adjusted for age group, sex and remoteness, and take into account the clustered nature of the data set. *P*_interaction_<0·05 indicates relationship between exposure and accessibility barriers is significantly different in urban/IR *v*. remote/OR areas.

* Variable significantly associated with accessibility barriers in remote/OR areas (*P* for Wald test <0·05).

† Variable significantly associated with accessibility barriers in urban/IR areas (*P* for Wald test <0·05).

‡ Significant difference in the relationship of the exposure to accessibility barriers between remote/OR and urban/IR carers (*P*_interaction_<0·05).

§ Inadequate data to assess *P*_interaction_.

#### Urban/inner regional areas

Although the absolute prevalence of accessibility barriers was much lower for carers in urban/IR *v*. remote/OR areas, we observed pronounced factors associated with accessibility barriers within the urban/IR group (Table 3). Advantage at both the family and area level was associated with a substantially lower prevalence of accessibility barriers. For example, the risk of accessibility barriers was reduced more than tenfold for urban/IR carers who reported they usually could save money *v*. ran out of money before the next payday (0·5 *v*. 6·7 %; PR=0·1, 95 % CI 0·0, 0·3) and who lived in areas with highest *v*. lowest tertile of advantage (≤1·2 *v*. 13·8 %; PR=0·1, 95 % CI 0·0, 0·3). We also observed a lower prevalence of accessibility barriers among carers who were partnered (1·9 *v*. 4·1 %; PR=0·4, 95 % CI 0·2, 0·8), who had weekly incomes ≥$AU 600 *v*. <$AU 600 (2·1 *v*. 4·5 %; PR=0·5, 95 % CI 0·3, 0·9) and who did not report worries about money (2·1 *v*. 4·9 %; PR=0·4, 95 % CI 0·2, 0·7) or experience food insecurity (2·4 *v*. 13·3 %; PR=0·3, 95 % CI 0·1, 0·8) in the past year.

Factors related to the sharing of resources were also significantly associated with accessibility barriers; carers were significantly less likely to report accessibility barriers if they were not humbugged in the past year (1·9 *v*. 6·0 %; PR=0·4, 95 % CI 0·2, 0·9) and if they were not pressured to support others in their community (1·6 *v*. 7·3 %; PR=0·3, 95 % CI 0·1, 0·6). Urban/IR carers were also less likely to report accessibility barriers if they did not experience issues with their housing (PR=0·3, 95 % CI 0·2, 0·7 for overcrowding; PR=0·2, 95 % CI 0·1, 0·4 for problems with cooking facilities; PR=0·4, 95 % CI 0·2, 0·9 for problems with home security).

Carers living in urban/IR areas were less likely to report accessibility barriers if they or their children had good social and emotional well-being, and if they lived in communities without racially motivated violence (2·1 *v*. 5·6 %; PR=0·2, 95 % CI 0·1, 0·6) or alcohol misuse (1·6 *v*. 4·9 %; PR=0·4, 95 % CI 0·2, 0·9). As observed in remote/OR areas, urban/IR carers who considered it important to teach their children about bush tucker were more likely to report accessibility barriers than carers who did not consider it important.

#### Similarities and differences in remote/outer regional v. urban/inner regional areas

In most cases, the relationship between exposures and accessibility barriers was consistent within the urban/IR and remote/OR samples, although the magnitudes of effect were often much larger for the urban/IR *v*. remote/OR group. However, we did observe that some exposures were differentially associated with accessibility barriers for carers in urban/IR compared with remote/OR environments (*P*_interaction_<0·05); this occurred for child and carer social and emotional well-being, carer’s relationship status, financial strain, worries about money, problems with racially motivated violence, overcrowding and problems with cooking facilities in the home. In all but one of these cases, we observed a significant association between the exposure and accessibility barriers in the urban/IR sample, but a null relationship in the remote/OR sample. In the case of racially motivated violence, the relationship was significant in both the urban/IR and remote/OR samples, but the effect size was markedly greater in the urban/IR sample. We did not calculate the *P* value for interaction between the dichotomous remoteness variable and the child’s Indigenous identification, given the small number of Torres Strait Islander children living in urban/IR areas.

### Factors associated with children’s dislike of fruits and vegetables

Among carers who did not report barriers related to accessibility, we observed few factors significantly associated with children’s dislike of fruit and vegetables (Table 4). After adjustment for age, sex and remoteness, carers were less likely to report dislike as a barrier if their children had good physical health and social and emotional well-being; the prevalence of dislike also varied by the employment status of the carer. Carers were significantly less likely to report dislike as a barrier if they lived in communities where there was no problem with alcohol use (32·7 *v*. 36·1 %; PR=0·9, 95 % CI 0·8, 1·0).

**Table 4:** Factors associated with children’s dislike of fruit and vegetable intake, among children whose carers who did not report accessibility barriers, in the Australian Longitudinal Study of Indigenous Children (LSIC), Wave 6, 2013

			Adjusted for age and sex		Adjusted for age, sex and remoteness	
	%	*n*/*N*	PR	95 % CI	PR	95 % CI
Total	34·2	390/1139	–	–	–	–
Child factors				
Sex						
Male	33·6	192/571	1·00		1·00	
Female	34·9	198/568	1·04	0·86, 1·25	1·04	0·86, 1·25
Age group						
4–5 years	33·7	85/252	1·00		1·00	
6–7 years	32·7	141/431	0·97	0·80, 1·17	0·98	0·81, 1·18
8–10 years	36·0	164/456	1·07	0·88, 1·29	1·07	0·88, 1·29
Indigenous identification						
Aboriginal	35·0	346/988	1·00		1·00	
Torres Strait Islander	27·4	23/84	0·78	0·58, 1·04	0·83	0·59, 1·16
Both	31·3	21/67	0·91	0·65, 1·27	0·92	0·65, 1·30
General physical health*,†						
Poor, fair, or good	40·7	103/253	1·00		1·00	
Very good or excellent	32·4	287/886	0·80	0·68, 0·94	0·77	0·65, 0·92
Social and emotional well-being*,†						
High risk of difficulties	39·2	96/245	1·00		1·00	
Low risk of difficulties	32·9	293/891	0·83	0·71, 0·98	0·83	0·71, 0·98
BMI category						
Overweight or obese	35·5	131/369	1·00		1·00	
Normal weight	35·2	184/523	1·00	0·85, 1·17	1·01	0·86, 1·19
Underweight	≤20·0	≤3/15	0·18	0·03, 1·10	0·21	0·03, 1·26
Family factors						
Carer’s general physical health						
Poor, fair, or good	33·9	216/637	1·00		1·00	
Very good or excellent	34·7	174/502	1·03	0·85, 1·24	1·02	0·84, 1·23
Carer’s social and emotional well-being						
High distress	38·2	84/220	1·00		1·00	
Low distress	33·3	303/909	0·87	0·73, 1·03	0·87	0·73, 1·03
Negative major life events in past year						
3–9	33·7	267/792	1·00		1·00	
<3	35·3	122/346	0·96	0·81, 1·14	0·96	0·81, 1·13
Carer is partnered						
No	31·2	159/509	1·00		1·00	
Yes	36·7	231/630	1·18	0·98, 1·42	1·18	0·98, 1·42
Weekly household income						
<$AU 600	33·4	129/386	1·00		1·00	
≥$AU 600	35·6	236/663	1·06	0·86, 1·31	1·05	0·85, 1·30
Financial strain						
Run out of money	34·7	41/118	1·00		1·00	
Just enough money	36·6	174/475	1·06	0·85, 1·31	1·08	0·87, 1·34
Can save money	31·7	171/540	0·92	0·73, 1·15	0·95	0·75, 1·19
Worries about money in past year						
Yes	31·2	96/308	1·00		1·00	
No	35·2	290/825	1·13	0·94, 1·36	1·15	0·96, 1·38
Went without meals in past year						
Yes	38·0	19/50	1·00		1·00	
No	33·9	367/1082	0·88	0·63, 1·25	0·87	0·61, 1·25
Carer’s employment status*,†						
Not employed	30·8	207/673	1·00		1·00	
Employed part-time	41·9	93/222	1·36	1·12, 1·64	1·34	1·11, 1·62
Employed full-time	35·1	78/222	1·14	0·90, 1·44	1·16	0·91, 1·46
Carer’s highest qualification						
Less than Year 12	33·1	193/583	1·00		1·00	
Year 12 and beyond	37·2	172/462	1·12	0·94, 1·33	1·12	0·93, 1·34
Humbugged in past year						
Yes	32·4	93/287	1·00		1·00	
No	34·7	295/850	1·07	0·90, 1·27	1·05	0·89, 1·24
Pressured to support others in the community						
Small or big problem	33·3	81/243	1·00		1·00	
Not a problem	34·0	271/797	1·02	0·80, 1·29	0·98	0·78, 1·24
Feed others who don’t live at home						
A few times a month or more	36·1	236/653	1·00		1·00	
Rarely or never	31·8	154/485	0·88	0·74, 1·04	0·89	0·75, 1·04
Had evening meal as a family in past week						
No	39·3	22/56	1·00		1·00	
Yes	33·9	365/1078	0·86	0·67, 1·11	0·84	0·65, 1·09
Cultural knowledge about bush tucker*						
Not important	36·8	289/785	1·00		1·00	
Somewhat important	30·7	73/238	0·83	0·66, 1·06	0·85	0·66, 1·10
Very important	23·5	27/115	0·64	0·45, 0·91	0·66	0·45, 0·95
Total number of people in household						
2–5	35·1	249/710	1·00		1·00	
≥6	32·9	141/429	0·94	0·80, 1·09	0·95	0·82, 1·11
House felt too crowded in past year						
Yes	39·7	52/131	1·00		1·00	
No	33·3	334/1002	0·84	0·67, 1·06	0·84	0·67, 1·04
Moved house in past year						
Yes	30·1	65/216	1·00		1·00	
No	35·0	321/917	1·16	0·93, 1·44	1·17	0·94, 1·46
Problem with fridge and/or cooking facilities						
Yes	35·3	18/51	1·00		1·00	
No	36·0	276/766	1·02	0·68, 1·52	0·98	0·65, 1·48
Electrical problems at home						
Yes	35·2	19/54	1·00		1·00	
No	34·0	368/1082	0·97	0·66, 1·42	0·98	0·67, 1·44
Security problems at home						
Yes	37·7	40/106	1·00		1·00	
No	33·7	347/1030	0·89	0·69, 1·17	0·88	0·68, 1·15
Area-level factors						
Racially motivated violence						
Small or big problem	36·0	58/161	1·00		1·00	
Not a problem	33·6	299/889	0·94	0·70, 1·25	0·97	0·72, 1·30
Alcohol misuse†						
Small or big problem	36·1	183/507	1·00		1·00	
Not a problem	32·7	196/599	0·91	0·78, 1·05	0·86	0·75, 1·00
Break-ins or theft						
Small or big problem	36·3	194/535	1·00		1·00	
Not a problem	31·4	171/545	0·86	0·73, 1·02	0·87	0·74, 1·02
Area-level disadvantage						
Least advantaged	30·6	45/147	1·00		1·00	
Mid-advantaged	33·5	246/734	1·09	0·82, 1·46	1·00	0·76, 1·33
Most advantaged	38·4	99/258	1·25	0·92, 1·72	1·18	0·84, 1·65
Remoteness						
None	35·2	120/341	1·00	–	–
Low	35·6	213/599	1·01	0·82, 1·24	–	–
Moderate	28·5	37/130	0·82	0·58, 1·14	–	–
High/extreme	29·0	20/69	0·81	0·62, 1·06	–	–

All models take into account the clustered nature of the data set.

* Variable significantly associated with dislike in model adjusted for age group and sex (*P* for Wald test <0·05).

† Variable significantly associated with dislike in model adjusted for age group, sex and remoteness (*P* for Wald test <0·05).

We did not observe significant variation in the prevalence of dislike by remoteness, or observe material changes in the relationship between exposures and dislike after additional adjustment for remoteness; thus, there was no indication for separate examination of these relationships in the remote/OR and urban/IR samples.

### Contextualisation

#### Accessibility barriers

Key informants provided insight into the meaning of carers’ reported barriers to children’s fruit and vegetable consumption and their relationship to child, family and community factors. Key informants explained that in remote/OR settings, low incomes, high food prices and pressures to support others compound to prevent carers from providing their children with fruit and vegetables despite awareness of the nutritional benefits:

‘A lot of people in the sort of remote communities are on benefits. And if you have got to feed a couple of kids … and you have to buy all of this fruit and veg, whereas you can have a couple packages of chips, or takeaway – well, what are you going to do? … Even though they may know it’s [fruit and veg] really good for them.’

Key informants explained how demand sharing influenced carers’ food purchasing behaviours, for example, discouraging carers from buying certain types of food:

‘Because if your child walks outside with watermelon, then you’ve got fifty kids all of a sudden knocking on your door.’

Given the existing barriers to purchasing healthy foods, key informants explained that health promotion efforts to increase children’s desire to eat fruit and vegetables have the unintended consequence of disempowering parents:

‘Because the kids do get, “You have to do this, have to do this”, go home and hassle Mum. “We need to do this, we need to do that”. And they’re [parents are] already under lots of pressure, stress. So, I think if they do those sorts of things [nutrition education], they’ve got to do something about the supply.’

Another key informant explained how families struggled to achieve the ‘Australian dream’ of providing a healthy diet for their children:

‘But what can you do? There is the pressure of education – you’ve got to send these kids to school, got to give them healthy lunches. Parents spend – but how far that $500 takes them; it’s not far. There is this health education; trying to live the Australian dream but you can’t.’

Key informants also described the limited availability and poor quality of fresh produce in remote/OR settings. For example, one key informant depicted the quality of fresh produce in remote/OR areas by asking, ‘Have you ever eaten frozen lettuce?’

Interviewers also commented that the competing affordability and availability of fast food was a barrier to the consumption of healthier foods; a key informant from an urban/IR setting explained that:

‘When somebody told me that veggies were too expensive back in my area, it would have been because Macca’s [McDonald’s] costs a lot less … it’s just that there is a cheaper alternative.’

Carers’ time scarcity was also considered an important barrier to nutrition, favouring quicker meal options such as takeaway.

#### Dislike

Key informants explained that children’s dislike of vegetables was a common problem, although many children were happy to eat fruit. One stated:

‘I came across this same complaint. That was, you cook it, and you prepare it, and they just don’t eat it.’

Key informants identified children’s lack of familiarity with some types of vegetables as an important contributor to their refusal to eat them:

‘It’s like introducing it to them … You give them a bowl of salad and some veggies, and they freak out; they just eat the meat and rice and they push the veggies aside. We tell them, “It’s good for you” but it sort of, it takes time to adjust.’

Key informants commented that ‘the expense of throwing away’ was a barrier to purchasing vegetables when there was a risk that children would refuse to eat them. They also explained that carers wanted to provide food that the child wanted to eat; carers’ food provision was more about ‘pleasing’ the child ‘than it is about what they’re feeding them at this stage’. The ubiquity of alternatives such as lollies and fast food, particularly in more urban areas, was considered to contribute to children’s picky eating and refusal to eat vegetables.

## Discussion

In this diverse national sample, almost half of carers reported barriers to their children’s nutrition. Barriers to fruit intake were less commonly reported than barriers to vegetable intake, but both are considered important nutritionally. Multiple barriers restrict Indigenous children’s consumption of fruit and vegetables. The most commonly reported barrier was children’s dislike of fruit and vegetables. Problems accessing fruit and vegetables were common among carers living in remote/OR settings and among disadvantaged carers living in urban/IR settings; the cost, availability and quality of fruit and vegetables were substantial barriers to access. Child and carer well-being, financial security, suitable housing and community cohesion promoted access to fruit and vegetables.

### Accessibility barriers

In this sample, carers were over ten times more likely to face problems accessing fruit and vegetables for their children if they lived in very remote areas compared with large cities. This is consistent with the increased price of fresh food in remote areas^(^ ^18^ ^)^ and the limited availability and poor quality of fresh foods, particularly after extended transportation^(^ ^17^ ^)^. These characteristics of the remote/OR food environment may explain why we observed few factors significantly associated with accessibility barriers within this group: where the environment itself creates substantial barriers to access, individual characteristics may become less important.

Within the urban/IR group, disadvantaged carers faced a tenfold increase in the relative risk of accessibility barriers compared with advantaged carers. Key informants described how the affordability and availability of fast-food outlets in urban/IR areas negatively influenced children’s consumption of fruit and vegetables. These findings are consistent with the literature^(^ ^26^ ^,^ ^54^ ^,^ ^55^ ^)^ and the increased availability of fast-food outlets in disadvantaged *v*. advantaged urban settings^(^ ^56^ ^,^ ^57^ ^)^. Additionally, some literature has also documented reduced accessibility of healthy foods in disadvantaged areas of Australia^(^ ^58^ ^)^, although findings are mixed^(^ ^15^ ^,^ ^57^ ^,^ ^59^ ^,^ ^60^ ^)^. Thus, for carers living in both remote/OR and disadvantaged urban/IR areas, the food environment constrained their ability to provide their child with a healthy diet, despite their nutritional awareness.

The present work provides quantitative evidence that multiple domains of financial security, including reported income, financial strain and worries about money, relate to carers’ ability to access fruit and vegetables for their children. These findings are consistent with evidenced socio-economic gradients in dietary intake; healthy diets tend to be more expensive than unhealthy diets^(^ ^22^ ^,^ ^61^ ^)^ and the literature describes an increased reliance on energy-dense foods (rather than nutrient-dense foods such as fruit and vegetables) by families with limited financial resources and time^(^ ^19^ ^,^ ^62^ ^,^ ^63^ ^)^.

Our findings provide quantitative evidence on the link between resource sharing and carers’ ability to purchase healthy foods. Consistent with previous qualitative findings^(^ ^12^ ^,^ ^17^ ^,^ ^21^ ^,^ ^27^ ^,^ ^64^ ^)^, our key informants explained that demand sharing encouraged the purchasing of energy-dense foods and discouraged the purchasing of sought-after foods that others are likely to request. Our findings also support the importance of adequate housing – in terms of crowding and household infrastructure – in promoting children’s nutrition^(^ ^9^ ^,^ ^21^ ^,^ ^65^ ^,^ ^66^ ^)^. Many Indigenous households contain extended family members rather than being restricted to the nuclear family, due to cultural differences in family structures and obligations to care for family. Lower socio-economic status and limited availability of quality housing (particularly in remote areas) further contribute to large numbers of household members, which is linked to the breakdown of housing infrastructure^(^ ^67^ ^,^ ^68^ ^)^.

Our study identified a relationship between access to fruit and vegetables and the social and emotional well-being of children and their carers. Key informants indicated that carers might experience distress or feel disempowered if they are unable to oblige their children’s requests for fruit and vegetables. These relationships may also be partially mediated by children’s and carer’s exposure to stressors and disadvantage. In both remote/OR and urban/IR settings, carers were more likely to report accessibility barriers if they considered it very important to pass on cultural knowledge about bush tucker. This might reflect that carers who value bush tucker reported barriers to accessing traditional fruit and vegetables or, alternatively, that carers who perceive accessibility barriers want to teach their children about bush tucker in order to supplement their diet.

We also observed a relationship between measures of community functioning (racially motivated violence and alcohol misuse) and carers’ ability to access fruit and vegetables for their children, providing evidence that community characteristics relate to children’s health^(^ ^39^ ^)^. The link between community-level racism and access to nutrition is consistent with wider literature linking racism to reduced access to resources required for health^(^ ^69^ ^,^ ^70^ ^)^. In communities where racism is perceived to be a problem, Indigenous people may be reluctant to go to food outlets; a qualitative study identified that some urban Indigenous people experiencing racism within their neighbourhood adopt an ‘avoidance strategy’^(^ ^71^ ^)^. The relationship between racism and accessibility barriers might be partially mediated by social and emotional well-being of the child and carer^(^ ^69^ ^,^ ^72^ ^,^ ^73^ ^)^ or by area-level disadvantage. Carers were also more likely to report barriers to accessing fruit and vegetables for their children if they perceived a problem with alcohol use in their community; carers may avoid shopping at food outlets where they perceive they may encounter alcohol-related issues, such as being asked for money to purchase alcohol.

#### Differences in remote/outer regional v. urban/inner regional areas

We generally observed consistent patterns in the associations between exposures and accessibility barriers in remote/OR and in urban/IR environments. However, there were some cases in which these relationships were statistically different. Some of the significant interactions observed might result from the high base prevalence of accessibility barriers in the remote/OR sample, which puts a ceiling on the possible magnitude of the relative risks within this group. This lack of potential for variation and the reduced sample size in the remote/OR sample limit the power to detect significant differences within the group. Although the relative risks observed within the remote/OR sample are often smaller in magnitude than those observed in the urban/IR sample, they are still associated with a large absolute difference in the proportion of carers experiencing the outcome (e.g. for financial strain and racially motivated violence). In addition, it is important to consider that some significant interactions could have resulted from multiple testing.

### Dislike

Where fruit and vegetables were accessible, children’s dislike was a substantial barrier to intake, indicating that it is important to consider multiple barriers when designing interventions to promote children’s fruit and vegetable consumption. Further, factors associated with dislike did not appear to be congruent with factors associated with accessibility barriers, suggesting that different population groups may face different barriers.

One-third of carers reported that children’s dislike was a barrier, with carers predominantly reporting an issue with dislike of vegetables, rather than fruit. These findings are consistent with comments by key informants, international literature on ‘picky eating’^(^ ^74^ ^)^ and a qualitative study on healthy eating among Aboriginal families in urban Victoria^(^ ^19^ ^)^. There is no accepted definition of picky eating, but it generally encompasses discomfort in eating familiar foods and/or unfamiliar foods^(^ ^75^ ^)^, and is considered to be associated with poorer diet quality, particularly an avoidance of vegetables, and increased intake of energy-dense snacks and sweets^(^ ^74^ ^)^. Internationally, the estimated prevalence of picky eating ranges from 6 to 50 %, with the peak prevalence among children aged 3 years (younger than our sample).

There is limited evidence on factors associated with picky eating. Research from the UK suggests a relationship between picky eating and socio-economic status^(^ ^74^ ^)^, but we did not observe a relationship between picky eating (dislike) and measures of socio-economic status in our sample, with the exception of carers’ employment status. This might have resulted from our restriction of the sample to carers who did not face accessibility problems, which removed some of the most disadvantaged families from the sample.

Our findings support research on the importance of familiarity and habit in shaping food choice^(^ ^17^ ^)^; key informants identified children’s lack of familiarity as an important contributor to their refusal to eat certain vegetables. The preference for familiar foods (such as those high in sugar and processed flour such as white bread) has been considered a lasting legacy of the colonial history that dramatically altered Indigenous food practices and preferences^(^ ^12^ ^,^ ^13^ ^,^ ^21^ ^,^ ^26^ ^,^ ^76^ ^)^.

The predominance of children’s dislike as a barrier to fruit and vegetable consumption is also consistent with the literature on traditional views of child autonomy and children’s ability to make their own choices^(^ ^17^ ^,^ ^64^ ^,^ ^77^ ^–^ ^79^ ^)^. This was supported by our key informants, who described the importance carers place on children’s own food preferences. Forcing children to eat fruit and vegetables conflicts with this cultural value; instead, carers are limited to influencing children’s food choice by encouraging them to consider healthy choices^(^ ^13^ ^,^ ^37^ ^,^ ^77^ ^,^ ^78^ ^,^ ^80^ ^–^ ^82^ ^)^.

Carers were less likely to report dislike for children with good physical health and social and emotional well-being, consistent with literature on the association between picky eating and physical, emotional and social impairment^(^ ^83^ ^)^. A strategy for overcoming children’s picky eating is to repeatedly expose (at least ten times) the child to the disliked food^(^ ^74^ ^,^ ^84^ ^)^. This approach is not feasible when the relevant foods are not readily available or are of poor quality, or when resources are scarce and there is concern about food wastage and hunger^(^ ^19^ ^,^ ^20^ ^)^, as mentioned by the key informants. Thus, although we have examined them separately, barriers related to dislike and to accessibility are likely to be entangled.

### Limitations

The current study relies predominantly on carer-reported data and findings should be interpreted as such. Our outcome reflects carers’ perceived barriers to their children’s nutrition, rather than actual barriers; however, previous research has demonstrated that perceived barriers are significantly correlated with intake^(^ ^85^ ^,^ ^86^ ^)^. We have validated that carers’ perception of barriers was closely tied to reported low intake by children, and that there was no material difference between the relationship of factors to low intake and to the perception of barriers. Further, our qualitative findings supported our quantitative findings, although we acknowledge that these qualitative findings were also based on the participants’ perceptions of these issues. A limitation of our outcome variable is that it would not serve as an indicator of a child’s nutrition status in cases where the child’s intake of fruit/vegetables exceeded recommended intake but the carer reported that they want their child to eat more, or in cases where the child’s intake of fruit/vegetables did not meet recommended intake but the carer reported that they do not want their child to eat more.

We recognise that there may be differences in the types of barriers reported, and the associated risk factors, in relation to fruit intake *v*. vegetable intake. For example, dislike is understood to be a more common barrier to vegetable intake than to fruit intake^(^ ^75^ ^)^. We have examined barriers to fruit and vegetable intake combined because fruit and vegetables play a similar nutritional role, and accessibility barriers for fruit and for vegetables are generally similar, as well as for pragmatic reasons.

The LSIC is not designed to be representative of the Australian Indigenous population; however, the LSIC data offer diversity in geography and environmental conditions and are well-suited for internal comparisons. Our study is based on cross-sectional data, so we cannot make inferences about causality. The potential biases arising from missing data should be minimal given that over 99 % (*n* 1230/1239) of carers participating in the survey provided data on the outcome and the majority of exposure variables had less than 1 % of data missing.

Grouping environments into discrete categories of remoteness is likely to have the effect of combining individuals living in areas across a range of remoteness, but the use of these categories is pragmatic and can define groups with similarities in life circumstances, such as the dominant social and cultural setting^(^ ^87^ ^)^.

## Conclusions

To our knowledge, the present study is the first large-scale investigation of barriers to Indigenous Australian children’s nutrition across heterogeneous environments. It provides quantitative evidence that a broad range of social, cultural and environmental factors impact nutrition. Disadvantaged Indigenous families in urban and inner regional settings, as well as those living in remote and outer regional areas, face substantial barriers to accessing fruit and vegetables. Where fruit and vegetables were accessible, dislike of vegetables was a common barrier.

Approaches to improve equity in nutrition in Australia need to address these barriers, through a combination of policy to improve the food environment in remote/OR and disadvantaged urban/IR settings and culturally relevant programmes to promote underlying determinants including financial security, housing and community cohesion^(^ ^22^ ^)^. Programmes and policies to promote nutrition should draw on the strengths of Indigenous families and communities, and must be conducted in partnership with Indigenous communities and individuals^(^ ^24^ ^,^ ^88^ ^)^.

## Supplementary material

For supplementary material accompanying this paper visit https://doi.org/10.1017/S1368980016003013. click here to view supplementary material
