# Supplementary material for: Understanding barriers to fruit and vegetable intake in the Australian Longitudinal Study of Indigenous Children: a mixed-methods approach
Source: Public Health Nutr. 2016 Nov 29;20(5):832–47. doi: 10.1017/S1368980016003013 (PMC5426329; doi:10.1017/S1368980016003013)
Supplement: Supplementary file 1 [file S1368980016003013sup001.docx]

# **Online supplementary material**

## Supplemental File 1

**CHILD FACTORS:**

Age at the time of survey was categorised as 4-5, 6-7, and 8-10 years.

General health: carers were asked, “In general, would you say <study child>’s health is excellent, very good, good, fair or poor?”; responses were categorised as poor, fair, or good vs. very good or excellent.

**Social and emotional wellbeing** was measured using the Strengths and Difficulties Questionnaire (SDQ; Copyright Robert Goodman, 1999, UK), designed for children over 3 years of age. Validation studies within Indigenous Australian populations have indicated that the Strengths and Difficulties Questionnaire is as an acceptable, internally consistent screening tool with good convergent validity^(^[^1^](#_ENREF_1)^,^ [^2^](#_ENREF_2)^)^.

Carers were prompted: “The next questions are about <study child>’s behaviour and how <he/she> gets along with other people. There are no right or wrong answers and every child is different”. Carers responded whether each of 25 statements was “Not true” (coded as 0), “Somewhat true” (coded as 1), or “Certainly true” (coded as 2) for 25 items^(^[^3^](#_ENREF_3)^)^. The SDQ measures children’s social and emotional behaviour across 5 domains: emotional symptoms, conduct problems, hyperactivity, peer problems, and prosocial behaviour. Scores are not calculated for children who are missing >2 questions within each sub-scale. We analysed the SDQ Total Difficulties Score, which summed scores across the first four subscales. The prosocial scale is intended to be analysed separately. Total Difficulty Scores are categorised as normal (0 to 13), or moderate (14 to 16) and high (17 to 40) risk of social and emotional difficulties^(^[^3^](#_ENREF_3)^,^ [^4^](#_ENREF_4)^)^.

**Weight status:** LSIC Research Administration Officers measured children’s height and weight using Homedics model SC-305-AOU-4209 digital scales and Soehnle professional Model 5003 stadiometers. These measurements were used to calculate Body Mass Index z-scores, specific to age and sex, using the World Health Organization international reference^(^[^5^](#_ENREF_5)^,^ [^6^](#_ENREF_6)^)^. Children’s weight status was categorised as underweight, normal weight, or overweight/obese according to defined cut-offs^(^[^7^](#_ENREF_7)^,^ [^8^](#_ENREF_8)^)^. To improve their validity, these data have been cleaned using an approach based on WHO standards and protocols^(^[^8^](#_ENREF_8)^)^, with additional exclusion of BMI z-score measurements that indicated an extreme increase or decrease in BMI between consecutive waves (associated with a change in BMI z-score ≥4).

**FAMILY FACTORS:**

**Weekly household income**: carers were asked, “How much money do you usually get from ALL SOURCES of income IN TOTAL (including that of your partner) AFTER deductions are taken out for TAX, quarantined payments etc.)?”, with response options ranging from <$100 per week to $2000 or more per week. We categorised income as <$600 per week vs. ≥$600 per week.

**Financial strain**: Carers were asked, “Which words best describe your family’s money situation?”, with possible response options: “we run out of money before payday”; “we are spending more money than we get”; “we have just enough money to get us through to the next payday”; “there’s some money left over each week but we just spend it”; “we can save a bit every now and then”; “we can save a lot”; or “Don’t know”. Responses were categorized as: run out of money (“we run out of money before payday” or “we are spending more money than we get”), just enough money (“we have just enough money to get us through to the next payday” or “there’s some money left over each week but we just spend it”), or can save money (“we can save a bit every now and then” or “we can save a lot”).

**Serious worries about money**: carers were asked, “In the last 12 months has your family had serious worries about money?”. Carers responded yes, no, or don’t know.

**Food insecurity**: carers were asked, “In the last 12 months, have any of these happened to you because you were short of money? Went without meals”. Carers responded yes, no, or don’t know.

**Carer’s highest qualification:** At the second wave of LSIC, the carers were asked to report their highest level of educational qualification. This question was repeated in subsequent waves for new primary carers only; we used the most recent information provided.

**Carer’s employment status:** carers were asked, “Do you have a job or are you on leave from a job?”. Carers were categorised as being employed if they responded: yes (one job only); yes, more than one job; or yes, but am currently on leave (e.g. Maternity leave, sick leave, etc.). Carers were categorised as not being employed if they responded: no; permanently unable to work; retired; unpaid working (e.g. volunteering); or other. Carers were also asked to report the number of hours worked in all jobs. If the carer reported being employed but reported doing 0 hours of work, they were recoded as not employed. Carers were coded as part-time if they worked >0 and ≤34 hours, and full-time if they worked >34 hours.

**Humbugging**: carers were asked, “In the last 12 months have you or your family been humbugged (harassed for money)?”. Carers responded yes, no, or don’t know.

**Feeding others:** carers were asked, “Lots of people share food. How often do people who don't live here, eat here?’ Responses were: everyday, four to six times a week, two to three times a week, once a week, once or twice a month, rarely, or never. Responses were categorised as either a few times a month or more, or as rarely or never.

**Pressures to support others:** carers were prompted, “Now I would like to ask you about problems that may affect the area where you live. This includes everybody (Indigenous and Non-Indigenous people) in your area. Tell me which of these you think are a problem in the area where you live: people being pressured to support others.” Carers reported if they perceived no problem (doesn’t happen here), a small problem (happens a bit of the time), a big problem (happens a lot of the time), or a very big problem (happens all the time) with each. Responses were categorised as not a problem (no problem) or small to big problem (small, big, or very big problem).

**Carers’ general health**: Carers were asked, “In general, would you say your health is excellent, very good, good, fair or poor?” Responses were categorised as poor, fair, or good vs. very good or excellent.

**Carers’ social and emotional wellbeing**: LSIC includes a set of seven questions that can capture subjective wellbeing^(^[^9^](#_ENREF_9)^)^. Unfortunately these seven questions do not capture positive aspects of emotional wellbeing, which is an important part of wellbeing. This social and emotional wellbeing index includes questions related to depression (including anger and impulsivity) and anxiety, and can provide an indication of the level of negative emotional wellbeing^(^[^9^](#_ENREF_9)^)^. Carers are asked if, in the last three months, they:

1. Have you stopped liking things that used to be fun?
2. Have you felt like everything is hard work (even little jobs are too much)?
3. Have you felt so worried that your stomach (tummy) has got upset?
4. Have you ever felt so worried it was hard to breathe?
5. Do you get angry or wild real quick?
6. Have you felt so sad that nothing could cheer you up? Not even your friends made you feel better.
7. Do you do silly things without thinking that you feel ashamed about the next day?

Response options were: never (or not much), little bit (or sometimes), fair bit, lots (or lots of times), or don’t know. The first two responses were coded as 0 (never of little bit) and the latter two as 1 (fair bit or lots)^(^[^10^](#_ENREF_10)^)^. Scores for the 7 questions were summed for a total of 0-7, with higher scores reflecting better social and emotional wellbeing. Carers were categorised as having a lower distress score (score 0-1) or a higher distress score (2-7).

**Negative major life events**: we examined whether families had experienced a set of 9 major negative life events in the past year. These included: carer losing a job, carer leaving the family, children being cared for by someone else for more than a week, very sick family member, death of close friend or family member, family member with drug or alcohol problem, assault or mugging of close family member, family member arrested or jailed or had problem with police, children upset by family arguments, or children scared by other people’s behaviour. We categorised the number of negative major life events as <3 or 3-9.

**Evening meal as a family**: carers were asked, “Did you or <Study Child>’s other family members do any of the following things with <Study Child> last week: Have dinner together as a family?” Responses were yes, no, or don’t know.

**Cultural knowledge about bush tucker**: carers were asked what elements of Indigenous culture they wanted to pass down to their children at their age. They were asked to selecting up to 5 answers from a list, in order of importance: finding bush tucker, hunting and fishing; knowing your country, where you are from; knowing your family history and heritage; singing, music and dancing; painting or weaving; knowing traditions and ceremonies; speaking your language; family networks; storytelling and yarning; having pride in your identity, knowing who you are; showing respect; spiritual beliefs; or other. Responses were recoded such that a carers’ first choice received a score of 5 (e.g. most important), decreasing to 1 for 5th choice and 0 for not selected.

Passing down cultural knowledge about finding bush tucker, hunting and fishing was categorised as not important (not picked by carer), somewhat important (3rd-5th choice), very important (1st-2nd choice).

**Household size**: The reported number of people living in the child’s household (ranging from 2-17) was categorised as 2-5 or ≥6.

**Moved house, overcrowded**: carers were asked, “In the last 12 months have you felt too crowded where you live, moved house, or had housing problems?” Carers responded yes, no, or don’t know. Those that responded yes indicated if they had felt too crowded, moved house, and/or had housing problems.

**Problems with fridge and/or cooking facilities**: in wave 5 of LSIC (collected in 2012), carers were asked whether their home had a working fridge, and a working stove, oven, or other cooking facilities. We recoded if, in Wave 5, carers reported that they a functioning cooking facilities and fridge, or problems with one or both. Because this question was not asked in Wave 6, we used responses from Wave 5, but recoded values to missing if the carers reported at Wave 6 that they had moved house in the past year.

**Major electrical problems or security problems at home**: carers were asked, “Does your home have any major things that need fixing?” If they responded yes, they were asked to specify “What things need fixing?”

Carers selected if they had any “major electrical problems”, and if they had any problems with “windows, doors, screens, or locks” (security problems).

**Racially-motivated violence, alcohol misuse, and break-ins or theft in the community**: carers were prompted, “Now I would like to ask you about problems that may affect the area where you live. This includes everybody (Indigenous and Non-Indigenous people) in your area. Tell me which of these you think are a problem in the area where you live.” Problems included: break-ins, robbery and theft; drinking too much grog; and racially-motivated violence; carers reported if they perceived no problem (doesn’t happen here), a small problem (happens a bit of the time), a big problem (happens a lot of the time), or a very big problem (happens all the time) with each. Responses were categorised as not a problem (no problem) or small to big problem (small, big, or very big problem). Carers were asked to report whether these were problems in their community; they were not asked to report on whether they or their family had personally been affected by these problems.

**REFERENCES**

[1] Williamson A, Redman S, Dadds M et al. (2010) Acceptability of an emotional and behavioural screening tool for children in Aboriginal Community Controlled Health Services in urban NSW. Australian and New Zealand Journal of Psychiatry. 44: 894-900.

[2] Williamson A, McElduff P, Dadds M et al. (2014) The Construct Validity of the Strengths and Difficulties Questionnaire for Aboriginal Children Living in Urban New South Wales, Australia. Australian Psychologist. 49: 163-70.

[3] Australian Government Department of Families Housing Community Services and Indigenous Affairs. (2012) The Longitudinal Study of Indigenous Children: Key summary report from Wave 3. Canberra: FaHCSIA.

[4] Goodman R. (2011) SDQ: Information for researchers and professionals about the Strengths & Difficulties Questionnaires. Youth in Mind.

[5] World Health Organization. (2012) WHO Anthro (version 3.2.2 January 2011) and macros. Child growth standards.

[6] Blossner M, Siyam A, Borghi E et al. (2009) WHO AnthroPlus for personal computers manual: software for assessing growth of the world's children and adolescents. Geneva, Switzerland: Department of Nutrition for Health and Development.

[7] De Onis M, Lobstein T. (2010) Defining obesity risk status in the general childhood population: Which cut-offs should we use? International Journal of Pediatric Obesity. 5: 458-60.

[8] Thurber K, Banks E, Banwell C. (2014) Approaches to maximising the accuracy of anthropometric data on children: review and empirical evaluation using the Australian Longitudinal Study of Indigenous Children. Public Health Research and Practice. 25: e2511407.

[9] Biddle N. (2011) An exploratory analysis of the Longitudinal Survey of Indigenous Children. CAEPR Working Paper No. 77/2011. Centre for Aboriginal Economic Policy Research.

[10] Australian Government Department of Families Housing Community Services and Indigenous Affairs. (2013) Appendix B - Study terminology and definitions. Footprints in Time The Longitudinal Study of Indigenous Children: Key Summary Report from Wave 4. Canberra.

## Supplemental File 2

Carers of children in the older cohort were asked to report on their child’s usual fruit and vegetable intake; they were asked, ‘how many serves of fruit/vegetables does [study child] usually eat each day?’, with response options of 1 serve, 2 serves, 3 serves, 4 serves, 5 serves, 6 serves or more, less than one serve, doesn’t eat fruit, other (please specify), and don’t know (see Fig. S1).

Fig. S1: Questions about children’s fruit and vegetable intake used in Wave 6 of LSIC


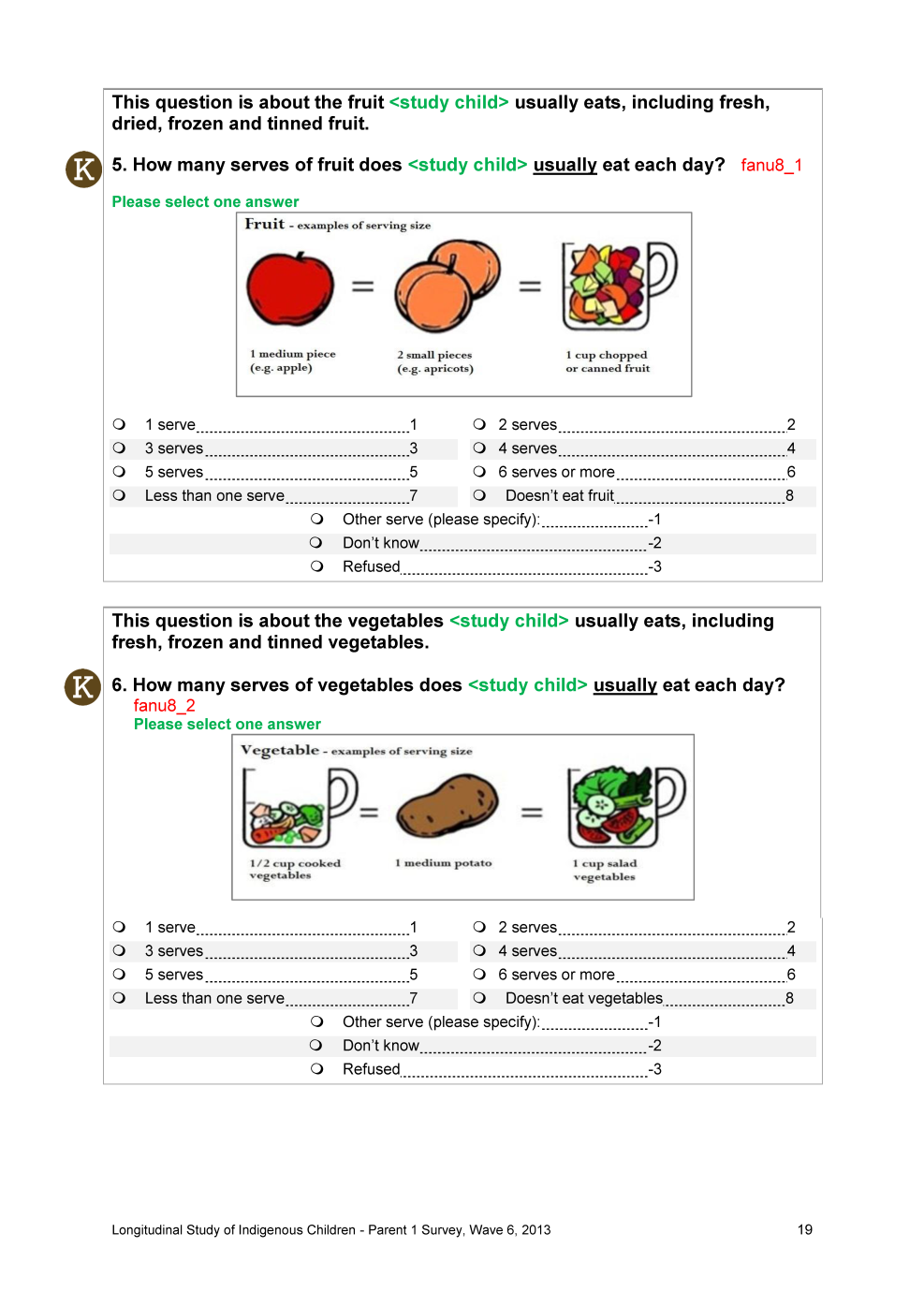


We observed that carers’ perception of barriers reflected low fruit and vegetable intake by their children (see Table S1). Reported average daily fruit consumption was significantly lower among children whose carers wanted them to eat more fruit (mean 1.39 serves per day, 95%CI:1.24,1.54), compared to children whose carers did not want them to eat more fruit (mean 2.45 serves per day, 95%CI:2.32,2.57). The same pattern was observed for vegetables; children whose carers wanted them to eat more vegetables reportedly consumed 1.43 servers of vegetables per day (95%CI:1.33,1.57), compared to 2.19 serves of vegetables per day (95%CI:2.05,2.33) for children whose carers did not want them to eat more vegetables. Thus, carers’ desire for children to eat more fruit and vegetables can indicate carers who face a barrier to their children eating more fruit and vegetables, and serve as a marker of children’s low intake.

Given potential differences in nutritional awareness by carers’ education or by remoteness, we repeated these analyses stratified by the carer’s highest qualification (less than Year 12 versus Year 12 or more), and by urban/IR vs. remote/OR status. The relationship between carers’ perception of barriers and children’s fruit and vegetable intake did not vary by carers’ education or by remoteness: within each group, we observed a similar relationship between carers’ perception of barriers and children’s fruit and vegetable intake (Table S1).

Although the majority of children met recommendations for fruit intake (1.5 serves for children aged 4-8 years and 2 serves for children 9-11 years), few children met recommendations for vegetable intake (4.5 serves for children aged 4-8 years and 5 serves for children 9-11 years). Among carers who did not want their children to eat more vegetables, the mean consumption was 2.19 serves of vegetables per day; only 6.47% (n=18/278) of these children met age-specific recommendations. Thus, intake of vegetables is still suboptimal for children whose carers do not perceive a barrier; however, we can identify carers with particularly low intake by examining carers who do perceive a barrier to their child’s intake.

Table S1: Fruit and vegetable intake among older children in LSIC Wave 6, by carers’ desire for children to eat more fruit and/or vegetables.

|  | **Mean number of usual daily serves of fruit** [95% CI] | | |  | **Mean number of usual daily serves of vegetables** [95% CI] | | |
| --- | --- | --- | --- | --- | --- | --- | --- |
|  | No barriers | Perceived any barrier | Total |  | No barriers | Perceived any barrier | Total |
|  |  |  |  |  |  |  |  |
| **Total older cohort** | 2.45 [2.32,2.57] | 1.39 [1.24,1.54] | 2.11 [2.00,2.22] |  | 2.19 [2.05,2.33] | 1.43 [1.33,1.57] | 1.86 [1.76,1.96] |
| (n) | (337) | (156) | (493) |  | (278) | (217) | (495) |
|  |  |  |  |  |  |  |  |
| **Carer’s highest qualification** |  |  |  |  |  |  |  |
| Less than Year 12 | 2.44 [2.26,2.62] | 1.33 [1.13,1.52] | 2.11 [1.95,2.26] |  | 2.24 [2.04,2.43] | 1.35 [1.14,1.56] | 1.87 [1.72,2.02] |
| (n) | (171) | (73) | (244) |  | (144) | (102) | (246) |
| Year 12 or more | 2.44 [2.26,2.63] | 1.41 [1.16,1.65] | 2.08 [1.91,2.24] |  | 2.09 [1.87,2.32] | 1.49 [1.28,1.69] | 2.08 [1.91,2.24] |
| (n) | (135) | (74) | (209) |  | (108) | (101) | (209) |
|  |  |  |  |  |  |  |  |
| **Remoteness** |  |  |  |  |  |  |  |
| Urban/IR | 2.46 [2.32,2.60] | 1.33 [1.16,1.51] | 2.11 [1.98,2.23] |  | 2.18 [2.02,2.34] | 1.44 [1.28,1.60] | 2.11 [1.98,2.23] |
| (n) | (271) | (123) | (394) |  | (224) | (172) | (394) |
| Remote/OR | 2.39 [2.13,2.66] | 1.61 [1.31,1.90] | 2.13 [1.92,2.35] |  | 2.22 [1.95,2.50] | 4.4 [1.11,1.69] | 1.85 [1.64,2.06] |
| (n) | (66) | (33) | (99) |  | (66) | (33) | (99) |

The sample includes those with non-missing data on variables of interest. Only carers of children in the older cohort were asked to report on the child’s usual fruit and vegetable intake.

We explored if the relationship of our exposures to carers’ perception of barriers was similar to the relationship of our exposures to children’s low intake of vegetables; consistency of these relationships would support our assumption that examining risk factors for barriers would provide insight into risk factors for children’s low intake of fruit and vegetables.

We focused on vegetable intake because there was more variation in intake between children, because fewer children met recommendations for intake, and because the qualitative data suggested that barriers were particularly predominant for vegetable, compared to fruit, intake. We categorised children as having low vegetable intake if their carer reported that they usually consumed <2 servings of vegetables per day, and categorised children as having high vegetable intake if their carer reported that they usually consumed ≥2 servings of vegetables per day.

We examined the proportion of carers reporting any barriers, and the proportion of carers reporting low vegetable intake, across categories of the exposure variables used in our main analysis. We calculated the Prevalence Ratios (Risk Ratios) for the outcome across exposure categories, using a multilevel Poisson model with robust variance. A variable to identify children’s geographic cluster was included as a level variable to account for the within-cluster correlation resulting from LSIC’s design. We adjusted these models first for age and sex only. We observed no material difference in the relationship between the exposures and the two outcomes (low vegetable intake and barriers), with the confidence intervals for the PRs for the two outcomes overlapping for each exposure group (Table S2).

**Table S2: Factors associated with low vegetable intake and carers’ perception of any barriers to intake, among children in the older cohort**

|  | **Older cohort** | | | | | | | | |
| --- | --- | --- | --- | --- | --- | --- | --- | --- | --- |
|  | **Perception of any barrier(s)** | | | |  | **Low vegetable intake** | | | |
|  | **%** | **(n/N)** | **PR** | **[95%CI]** |  | **%** | **(n/N)** | **PR** | **[95%CI]** |
| **TOTAL** | 49.3 | (244/495) | -- | -- |  | 45.5 | (227/499) | -- | -- |
| **CHILD FACTORS** |  |  |  |  |  |  |  |  |  |
| **Sex *** |  |  |  |  |  |  |  |  |  |
| Male | 50.8 | (124/244) | 1 |  |  | 50.8 | (124/244) | 1 |  |
| Female | 47.8 | (120/251) | 0.94 | [0.78,1.13] |  | 40.6 | (102/251) | 0.81 | [0.69,0.97] |
| **Age group** |  |  |  |  |  |  |  |  |  |
| 4-5 years | -- | -- | -- | -- |  | -- | -- | -- | -- |
| 6-7 years | 57.1 | (4/7) | 1.16 | [0.62,2.15] |  | 57.1 | (4/7) | 1.25 | [0.70,2.23] |
| 8-10 years | 49.2 | (240/488) | 1 |  |  | 45.5 | (222/488) | 1 |  |
| **Indigenous identification** |  |  |  |  |  |  |  |  |  |
| Aboriginal | 50.7 | (220/434) | 1 |  |  | 44.9 | (195/434) | 1 |  |
| Torres Strait Islander | 39.5 | (15/38) | 0.78 | [0.55,1.11] |  | 47.4 | (18/38) | 1.08 | [0.72,1.62] |
| Both | 39.1 | (9/23) | 0.77 | [0.48,1.26] |  | 56.5 | (13/23) | 1.25 | [0.84,1.85] |
| **General physical health †** |  |  |  |  |  |  |  |  |  |
| Poor, fair, or good | 59.0 | (79/134) | 1 |  |  | 45.5 | (61/134) | 1 |  |
| Very good or excellent | 45.7 | (165/361) | 0.77 | [0.65,0.93] |  | 45.7 | (165/361) | 1.00 | [0.82,1.22] |
| **Social and emotional wellbeing** |  |  |  |  |  |  |  |  |  |
| High risk of difficulties | 48.6 | (53/109) | 1 |  |  | 51.4 | (56/109) | 1 |  |
| Low risk of difficulties | 49.2 | (189/384) | 1.02 | [0.83,1.26] |  | 44.3 | (170/384) | 0.90 | [0.74,1.08] |
| **BMI category †** |  |  |  |  |  |  |  |  |  |
| Overweight or obese | 46.1 | (76/165) | 1 |  |  | 40.6 | (67/165) | 1 |  |
| Normal weight | 56.4 | (123/218) | 1.23 | [1.00,1.51] |  | 46.8 | (102/218) | 1.13 | [0.88,1.45] |
| Underweight | ≤27.3 | (≤3/11) | 0.39 | [0.10,1.50] |  | 63.6 | (7/11) | 1.57 | [0.94,2.64] |
| **FAMILY FACTORS** |  |  |  |  |  |  |  |  |  |
| **Carer's general physical health** |  |  |  |  |  |  |  |  |  |
| Poor, fair, or good | 49.8 | (148/297) | 1 |  |  | 45.1 | (134/297) | 1 |  |
| Very good or excellent | 48.5 | (96/198) | 0.97 | [0.81,1.16] |  | 46.5 | (92/198) | 1.05 | [0.86,1.28] |
| **Carer's social and emotional wellbeing** |  |  |  |  |  |  |  |  |  |
| High distress | 55.4 | (51/92) | 1 |  |  | 52.2 | (48/92) | 1 |  |
| Low distress | 47.9 | (191/399) | 0.86 | [0.70,1.07] |  | 44.1 | (176/399) | 0.86 | [0.67,1.11] |
| **Negative major life events in past year** |  |  |  |  |  |  |  |  |  |
| 3-9 | 48.3 | (83/172) | 1 |  |  | 43.0 | (74/172) | 1 |  |
| <3 | 49.8 | (161/323) | 1.04 | [0.80,1.34] |  | 47.1 | (152/323) | 1.11 | [0.88,1.40] |
| **Carer is partnered** |  |  |  |  |  |  |  |  |  |
| No | 47.8 | (110/230) | 1 |  |  | 48.3 | (111/230) | 1 |  |
| Yes | 50.6 | (134/265) | 1.06 | [0.87,1.28] |  | 43.4 | (115/265) | 0.89 | [0.72,1.09] |
| **Weekly household income** |  |  |  |  |  |  |  |  |  |
| <$600 | 49.7 | (81/163) | 1 |  |  | 46.6 | (76/163) | 1 |  |
| ≥$600-$999 | 49.0 | (141/288) | 0.99 | [0.80,1.22] |  | 44.8 | (129/288) | 0.96 | [0.75,1.22] |
| **Financial strain** |  |  |  |  |  |  |  |  |  |
| Run out of money | 50.9 | (29/57) | 1 |  |  | 38.6 | (22/57) | 1 |  |
| Just enough money | 53.3 | (114/214) | 1.06 | [0.83,1.34] |  | 44.9 | (96/214) | 1.21 | [0.81,1.83] |
| Can save money | 44.8 | (100/223) | 0.89 | [0.70,1.13] |  | 48.0 | (107/223) | 1.27 | [0.83,1.95] |
| **Worried about money in past year** |  |  |  |  |  |  |  |  |  |
| Yes | 45.4 | (64/141) | 1 |  |  | 44.0 | (62/141) | 1 |  |
| No | 51.0 | (179/351) | 1.13 | [0.91,1.40] |  | 46.4 | (163/351) | 1.09 | [0.87,1.38] |
| **Went without meals in past year** |  |  |  |  |  |  |  |  |  |
| Yes | 52.4 | (11/21) | 1 |  |  | 47.6 | (10/21) | 1 |  |
| No | 49.1 | (231/470) | 0.94 | [0.62,1.41] |  | 45.3 | (213/470) | 0.97 | [0.61,1.53] |
| **Carer's employment status** |  |  |  |  |  |  |  |  |  |
| Not employed | 44.3 | (116/262) | 1 |  |  | 43.9 | (115/262) | 1 |  |
| Employed part-time | 56.1 | (64/114) | 1.26 | [1.05,1.53] |  | 50.0 | (57/114) | 1.11 | [0.87,1.42] |
| Employed full-time | 50.9 | (56/110) | 1.14 | [0.92,1.42] |  | 45.5 | (50/110) | 1.01 | [0.78,1.30] |
| **Carer’s highest qualification †** |  |  |  |  |  |  |  |  |  |
| Less than Year 12 | 46.3 | (114/246) | 1 |  |  | 47.2 | (116/246) | 1 |  |
| Year 12 and beyond | 55.0 | (115/209) | 1.18 | [1.02,1.38] |  | 46.9 | (98/209) | 0.98 | [0.80,1.18] |
| **Humbugged in past year** |  |  |  |  |  |  |  |  |  |
| Yes | 50.7 | (70/138) | 1 |  |  | 45.7 | (63/138) | 1 |  |
| No | 48.7 | (174/357) | 0.97 | [0.81,1.15] |  | 45.7 | (163/357) | 1.03 | [0.80,1.31] |
| **Pressured to support others in the community** |  |  |  |  |  |  |  |  |  |
| Small or big problem | 55.7 | (64/115) | 1 |  |  | 43.5 | (50/115) | 1 |  |
| Not a problem | 48.0 | (160/333) | 0.86 | [0.68,1.10] |  | 43.8 | (146/333) | 1.01 | [0.78,1.33] |
| **Feed others who don’t live at home** |  |  |  |  |  |  |  |  |  |
| A few times a month or more | 51.9 | (149/287) | 1 |  |  | 46.3 | (133/287) | 1 |  |
| Rarely or never | 45.7 | (95/208) | 0.88 | [0.72,1.08] |  | 44.7 | (93/208) | 0.97 | [0.78,1.19] |
| **Had dinner as a family in past week *** |  |  |  |  |  |  |  |  |  |
| No | 58.3 | (14/24) | 1 |  |  | 62.5 | (15/24) | 1 |  |
| Yes | 48.8 | (229/469) | 0.83 | [0.67,1.03] |  | 44.6 | (209/469) | 0.71 | [0.51,1.00] |
| **Cultural knowledge about bush tucker** |  |  |  |  |  |  |  |  |  |
| Not important | 51.1 | (168/329) | 1 |  |  | 46.5 | (153/329) | 1 |  |
| Somewhat important | 43.6 | (48/110) | 0.85 | [0.68,1.06] |  | 39.1 | (43/110) | 0.83 | [0.65,1.07] |
| Very important | 50.0 | (28/56) | 0.98 | [0.72,1.33] |  | 53.6 | (30/56) | 1.15 | [0.84,1.58] |
| **Total number of people in household *** |  |  |  |  |  |  |  |  |  |
| 2-5 | 52.3 | (159/304) | 1 |  |  | 51.6 | (157/304) | 1 |  |
| ≥6 | 44.5 | (85/191) | 0.85 | [0.73,1.00] |  | 36.1 | (69/191) | 0.71 | [0.57,0.88] |
| **House felt too crowded in past year** |  |  |  |  |  |  |  |  |  |
| Yes | 53.2 | (33/62) | 1 |  |  | 40.3 | (25/62) | 1 |  |
| No | 48.6 | (210/432) | 0.91 | [0.69,1.21] |  | 46.5 | (201/432) | 1.16 | [0.85,1.58] |
| **Moved house in past year** |  |  |  |  |  |  |  |  |  |
| Yes | 42.2 | (35/83) | 1 |  |  | 39.8 | (33/83) | 1 |  |
| No | 50.6 | (208/411) | 1.20 | [0.93,1.55] |  | 47.0 | (193/411) | 1.19 | [0.92,1.53] |
| **Problem with fridge and/or cooking facilities** |  |  |  |  |  |  |  |  |  |
| Yes | 52.0 | (13/25) | 1 |  |  | 32.0 | (8/25) | 1 |  |
| No | 50.7 | (174/343) | 0.98 | [0.71,1.35] |  | 48.4 | (166/343) | 1.52 | [0.81,2.85] |
| **Electrical problems at home** |  |  |  |  |  |  |  |  |  |
| Yes | 48.5 | (16/33) | 1 |  |  | 39.4 | (13/33) | 1 |  |
| No | 49.2 | (227/461) | 1.02 | [0.66,1.59] |  | 46.0 | (212/461) | 1.19 | [0.75,1.87] |
| **Security problems at home** |  |  |  |  |  |  |  |  |  |
| Yes | 56.1 | (32/57) | 1 |  |  | 43.9 | (25/57) | 1 |  |
| No | 48.3 | (211/437) | 0.86 | [0.63,1.18] |  | 45.8 | (200/437) | 1.06 | [0.77,1.47] |
| **AREA-LEVEL FACTORS** |  |  |  |  |  |  |  |  |  |
| **Racially-motivated violence** |  |  |  |  |  |  |  |  |  |
| Small or big problem | 51.9 | (42/81) | 1 |  |  | 49.4 | (40/81) | 1 |  |
| Not a problem | 49.2 | (188/382) | 0.94 | [0.72,1.24] |  | 43.2 | (165/382) | 0.88 | [0.66,1.18] |
| **Alcohol misuse †** |  |  |  |  |  |  |  |  |  |
| Small or big problem | 53.5 | (121/226) | 1 |  |  | 50.0 | (113/226) | 1 |  |
| Not a problem | 44.7 | (113/253) | 0.82 | [0.69,0.99] |  | 40.7 | (103/253) | 0.80 | [0.63,1.02] |
| **Break-ins or theft** |  |  |  |  |  |  |  |  |  |
| Small or big problem | 51.3 | (118/230) | 1 |  |  | 48.7 | (112/230) | 1 |  |
| Not a problem | 46.8 | (111/237) | 0.91 | [0.74,1.11] |  | 41.8 | (99/237) | 0.86 | [0.69,1.07] |
| **Area-level disadvantage** |  |  |  |  |  |  |  |  |  |
| Least advantaged | 56.4 | (44/78) | 1.0 |  |  | 47.4 | (37/78) | 1 |  |
| Mid-advantaged | 48.0 | (153/319) | 0.84 | [0.63,1.14] |  | 45.1 | (144/319) | 0.92 | [0.65,1.31] |
| Most advantaged | 48.0 | (47/98) | 0.85 | [0.62,1.17] |  | 45.9 | (45/98) | 0.96 | [0.67,1.37] |
| **Remoteness** |  |  |  |  |  |  |  |  |  |
| None | 50.7 | (68/134) | 1 |  |  | 53.0 | (71/134) | 1 |  |
| Low | 49.6 | (130/262) | 0.98 | [0.79,1.21] |  | 43.9 | (115/262) | 0.82 | [0.61,1.12] |
| Moderate | 36.0 | (18/50) | 0.70 | [0.43,1.14] |  | 38.0 | (19/50) | 0.70 | [0.47,1.03] |
| High/extreme | 57.1 | (28/49) | 1.14 | [0.81,1.61] |  | 42.9 | (21/49) | 0.85 | [0.56,1.29] |

The sample includes those with data on both outcomes, and the exposure of interest. All models are adjusted for age group and sex, and take into account the clustered nature of the dataset.

* Variable significantly associated with children’s low vegetable intake (p-value for Wald test <0.05).

† Variable significantly associated with carers reporting any barriers (p-value for Wald test <0.05).

## Supplemental File 3

Table S3: Factors associated with accessibility barriers to children’s fruit and vegetable intake

|  | **Accessibility barriers** | | | | | | | |
| --- | --- | --- | --- | --- | --- | --- | --- | --- |
|  |  |  |  | **Adjusted for age and sex** | |  | **Adjusted for age, sex, and remoteness** | |
|  | **%** | **(n/N)** |  | **PR** | **[95%CI]** |  | **PR** | **[95%CI]** |
| **TOTAL** | 7.4 | (91/1,230) |  | -- | -- |  | -- | -- |
| **CHILD FACTORS** |  |  |  |  |  |  |  |  |
| **Sex** |  |  |  |  |  |  |  |  |
| Male | 7.3 | (45/616) |  | 1 |  |  | 1 |  |
| Female | 7.5 | (46/614) |  | 0.85 | [0.60,1.21] |  | 0.85 | [0.61,1.20] |
| **Age group** |  |  |  |  |  |  |  |  |
| 4-5 years | 8.4 | (23/275) |  | 1 |  |  | 1 |  |
| 6-7 years | 7.7 | (36/467) |  | 0.93 | [0.62,1.41] |  | 0.9 | [0.59,1.37] |
| 8-10 years | 6.6 | (32/488) |  | 0.8 | [0.55,1.16] |  | 0.78 | [0.53,1.16] |
| **Indigenous identification** |  |  |  |  |  |  |  |  |
| Aboriginal | 7.7 | (82/1070) |  | 1 |  |  | 1 |  |
| Torres Strait Islander | ≤3.4 | (≤3/87) |  | 0.8 | [0.21,3.14] |  | 0.34 | [0.08,1.54] |
| Both | 8.2 | (6/73) |  | 1.33 | [0.57,3.11] |  | 0.76 | [0.31,1.87] |
| **General physical health *^†^** |  |  |  |  |  |  |  |  |
| Poor, fair, or good | 16.8 | (51/304) |  | 1 |  |  | 1 |  |
| Very good or excellent | 4.3 | (40/926) |  | 0.58 | [0.38,0.89] |  | 0.65 | [0.43,0.96] |
| **Social and emotional wellbeing *^†^** |  |  |  |  |  |  |  |  |
| High risk of difficulties | 12.8 | (36/281) |  | 1 |  |  | 1 |  |
| Low risk of difficulties | 5.8 | (55/946) |  | 0.61 | [0.39,0.93] |  | 0.59 | [0.38,0.92] |
| **Body Mass Index category** |  |  |  |  |  |  |  |  |
| Overweight or obese | 5.6 | (22/391) |  | 1 |  |  | 1 |  |
| Normal weight | 9.0 | (52/575) |  | 1 | [0.71,1.41] |  | 0.94 | [0.67,1.30] |
| Underweight | ≤16.7 | (≤3/18) |  | 1.01 | [0.36,2.82] |  | 0.83 | [0.30,2.31] |
| **FAMILY FACTORS** |  |  |  |  |  |  |  |  |
| **Carer's general physical health** |  |  |  |  |  |  |  |  |
| Poor, fair, or good | 9.6 | (68/705) |  | 1 |  |  | 1 |  |
| Very good or excellent | 4.2 | (22/524) |  | 0.65 | [0.39,1.06] |  | 0.67 | [0.40,1.11] |
| **Carer's social and emotional wellbeing** | |  |  |  |  |  |  |  |
| High distress | 9.1 | (22/242) |  | 1 |  |  | 1 |  |
| Low distress | 7.1 | (69/978) |  | 0.79 | [0.50,1.27] |  | 0.75 | [0.46,1.20] |
| **Negative major life events in past year ^†^** | |  |  |  |  |  |  |  |
| 3-9 | 10.6 | (41/387) |  | 1 |  |  | 1 |  |
| <3 | 5.9 | (50/842) |  | 0.77 | [0.59,1.01] |  | 0.76 | [0.58,0.99] |
| **Carer is partnered ^†^** |  |  |  |  |  |  |  |  |
| No | 7.3 | (40/549) |  | 1 |  |  | 1 |  |
| Yes | 7.5 | (51/681) |  | 0.76 | [0.52,1.11] |  | 0.76 | [0.52,1.11] |
| **Weekly household income *^†^** |  |  |  |  |  |  |  |  |
| <$600 | 13.6 | (61/447) |  | 1 |  |  | 1 |  |
| ≥$600 | 3.2 | (22/685) |  | 0.55 | [0.34,0.88] |  | 0.59 | [0.36,0.94] |
| **Financial strain †** |  |  |  |  |  |  |  |  |
| Run out of money | 15.1 | (21/139) |  | 1 |  |  | 1 |  |
| Just enough money | 8.8 | (46/521) |  | 0.74 | [0.46,1.21] |  | 0.71 | [0.43,1.17] |
| Can save money | 4.3 | (24/564) |  | 0.43 | [0.22,0.85] |  | 0.36 | [0.17,0.77] |
| **Worried about money in past year** | |  |  |  |  |  |  |  |
| Yes | 6.9 | (23/331) |  | 1 |  |  | 1 |  |
| No | 7.6 | (68/893) |  | 0.85 | [0.58,1.25] |  | 0.75 | [0.50,1.12] |
| **Went without meals in past year *^†^** | |  |  |  |  |  |  |  |
| Yes | 30.6 | (22/72) |  | 1 |  |  | 1 |  |
| No | 6.0 | (69/1151) |  | 0.50 | [0.32,0.78] |  | 0.49 | [0.31,0.77] |
| **Carer’s employment status** |  |  |  |  |  |  |  |  |
| Not employed | 8.4 | (62/735) |  | 1 |  |  | 1 |  |
| Employed part-time | 6.7 | (16/238) |  | 0.88 | [0.59,1.31] |  | 0.92 | [0.62,1.36] |
| Employed full-time | 5.1 | (12/234) |  | 0.74 | [0.47,1.18] |  | 0.71 | [0.44,1.15] |
| **Carer’s highest qualification** |  |  |  |  |  |  |  |  |
| Less than Year 12 | 9.0 | (58/641) |  | 1 |  |  | 1 |  |
| Year 12 and beyond | 5.9 | (29/491) |  | 0.95 | [0.67,1.35] |  | 0.95 | [0.67,1.34] |
| **Humbugged in past year *†** |  |  |  |  |  |  |  |  |
| Yes | 15.1 | (51/338) |  | 1 |  |  | 1 |  |
| No | 4.5 | (40/890) |  | 0.52 | [0.33,0.83] |  | 0.53 | [0.34,0.83] |
| **Pressured to support others in the community *^†^** | | |  |  |  |  |  |  |
| Small or big problem | 18.5 | (55/298) |  | 1 |  |  | 1 |  |
| Not a problem | 3.5 | (29/826) |  | 0.39 | [0.20,0.74] |  | 0.46 | [0.25,0.83] |
| **Feed others who don’t live at home** | |  |  |  |  |  |  |  |
| A few times a month or more | 8.4 | (60/713) |  | 1 |  |  | 1 |  |
| Rarely or never | 5.8 | (30/515) |  | 0.81 | [0.59,1.11] |  | 0.81 | [0.58,1.12] |
| **Had evening meal as a family in past week *** | |  |  |  |  |  |  |  |
| No | 8.2 | (5/61) |  | 1 |  |  | 1 |  |
| Yes | 7.4 | (86/1164) |  | 0.49 | [0.26,0.92] |  | 0.61 | [0.32,1.14] |
| **Cultural knowledge about bush tucker *^†^** | |  |  |  |  |  |  |  |
| Not important | 3.1 | (25/810) |  | 1 |  |  | 1 |  |
| Somewhat important | 11.9 | (32/270) |  | 1.9 | [1.16,3.12] |  | 1.43 | [0.88,2.33] |
| Very important | 22.8 | (34/149) |  | 3.08 | [1.74,5.44] |  | 2.34 | [1.38,3.99] |
| **Total number of people in household** | |  |  |  |  |  |  |  |
| 2-5 | 5.3 | (40/750) |  | 1 |  |  | 1 |  |
| ≥6 | 10.6 | (51/480) |  | 1.07 | [0.68,1.69] |  | 1.02 | [0.64,1.62] |
| **House felt too crowded in past year** | |  |  |  |  |  |  |  |
| Yes | 12.7 | (19/150) |  | 1 |  |  | 1 |  |
| No | 6.6 | (71/1073) |  | 0.7 | [0.36,1.38] |  | 0.68 | [0.33,1.36] |
| **Moved house in past year** |  |  |  |  |  |  |  |  |
| Yes | 7.3 | (17/233) |  | 1 |  |  | 1 |  |
| No | 7.4 | (73/990) |  | 1.03 | [0.73,1.44] |  | 0.99 | [0.70,1.39] |
| **Problem with fridge and/or cooking facilities ^†^** | | |  |  |  |  |  |  |
| Yes | 21.5 | (14/65) |  | 1 |  |  | 1 |  |
| No | 5.4 | (44/810) |  | 0.6 | [0.35,1.03] |  | 0.62 | [0.35,1.10] |
| **Electrical problems at home *†** |  |  |  |  |  |  |  |  |
| Yes | 21.7 | (15/69) |  | 1 |  |  | 1 |  |
| No | 6.6 | (76/1158) |  | 0.66 | [0.48,0.91] |  | 0.63 | [0.45,0.90] |
| **Security problems at home *^†^** |  |  |  |  |  |  |  |  |
| Yes | 21.5 | (29/135) |  | 1 |  |  | 1 |  |
| No | 5.7 | (62/1092) |  | 0.63 | [0.41,0.96] |  | 0.63 | [0.41,0.96] |
| **AREA-LEVEL FACTORS** |  |  |  |  |  |  |  |  |
| **Racially-motivated violence *†** |  |  |  |  |  |  |  |  |
| Small or big problem | 9.0 | (16/177) |  | 1 |  |  | 1 |  |
| Not a problem | 7.1 | (68/957) |  | 0.55 | [0.31,0.95] |  | 0.5 | [0.27,0.91] |
| **Alcohol misuse *^†^** |  |  |  |  |  |  |  |  |
| Small or big problem | 12.4 | (72/579) |  | 1 |  |  | 1 |  |
| Not a problem | 2.8 | (17/616) |  | 0.39 | [0.22,0.70] |  | 0.46 | [0.27,0.80] |
| **Break-ins or theft** |  |  |  |  |  |  |  |  |
| Small or big problem | 8.9 | (52/587) |  | 1 |  |  | 1 |  |
| Not a problem | 6.2 | (36/581) |  | 0.79 | [0.56,1.12] |  | 0.77 | [0.55,1.08] |
| **Area-level disadvantage *^†^** |  |  |  |  |  |  |  |  |
| Least advantaged | 31.6 | (68/215) |  | 1 |  |  | 1 |  |
| Mid-advantaged | 2.7 | (20/754) |  | 0.12 | [0.06,0.24] |  | 0.18 | [0.07,0.45] |
| Most advantaged | ≤1.1 | (≤3/261) |  | 0.05 | [0.01,0.15] |  | 0.08 | [0.02,0.33] |
| **Remoteness *** |  |  |  |  |  |  |  |  |
| None | 2.0 | (7/348) |  | 1 |  |  | -- | -- |
| Low | 3.4 | (21/620) |  | 1.62 | [0.55,4.82] |  | -- | -- |
| Moderate | 17.7 | (28/158) |  | 7.11 | [2.18,23.22] |  | -- | -- |
| High/extreme | 33.7 | (35/104) |  | 14.1 | [4.26,46.42] |  | -- | -- |

All models take into account the clustered nature of the dataset.

* Variable significantly associated with accessibility barriers in model adjusted for age group and sex (p-value for Wald test <0.05).

† Variable significantly associated with accessibility barriers in model adjusted for age group, sex, and remoteness (p-value for Wald test <0.05).
